# Nanoimprint Lithography as a Route to Nanoscale Back-Contact Perovskite Solar Cells

**DOI:** 10.1021/acsanm.3c02493

**Published:** 2023-08-16

**Authors:** Jonathon Harwell, Ifor D. W. Samuel

**Affiliations:** School of Physics and Astronomy, University of St Andrews, North Haugh, St Andrews KY16 9SS, United Kingdom

**Keywords:** perovskite, solar cell, back-contact, nanoimprint, lithography, nanoscale, interdigitated

## Abstract

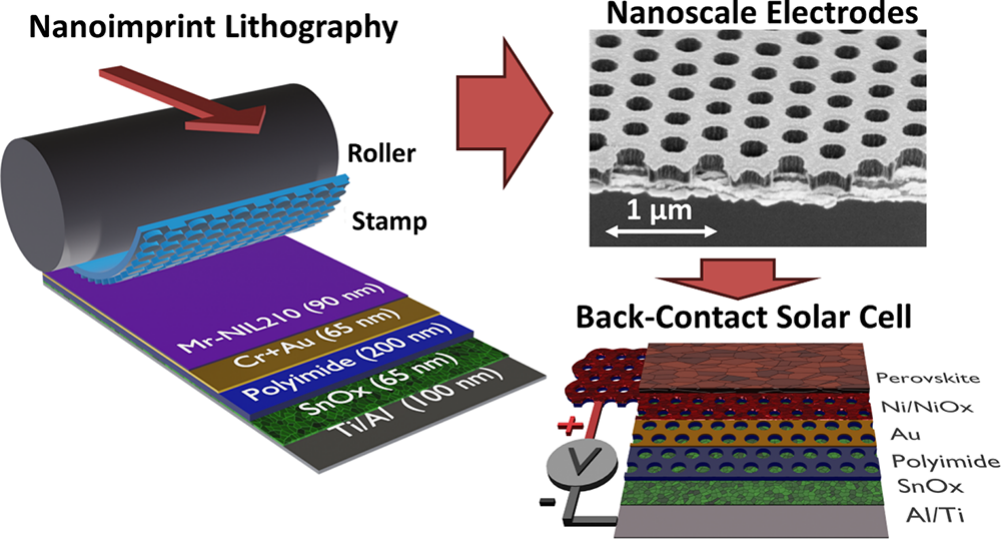

Back-contact perovskite solar cells are of great interest because they could achieve higher performance than conventional designs while also eliminating the need for transparent conductors. Current research in this field has focused on making electrode structures with reduced widths to collect charges more efficiently, but current lift-off-based fabrication techniques have struggled to achieve electrode widths smaller than 1000 nm and are difficult to implement on large areas. We demonstrate nanoimprint lithography in an etch-down procedure as a simple and easily scalable method to produce honeycomb-shaped, quasi-interdigitated electrode structures with widths as small as 230 nm. We then use electrodeposition to selectively deposit conformal coatings of a range of different hole-selective layers and explore how the efficiency of back-contact perovskite solar cells changes as the feature sizes are pushed into the nanoscale. We find that the efficiency of the resulting devices remains almost unchanged as the electrode width is varied from 230 to 2000 nm, which differs from reported device simulations. Our results suggest that reducing recombination and improving the quality of the charge transport layers, rather than reducing the minimum feature size, are likely to be the best pathway to maximizing the performance of back-contact perovskite solar cells.

## 1 Introduction

**Figure 1:**
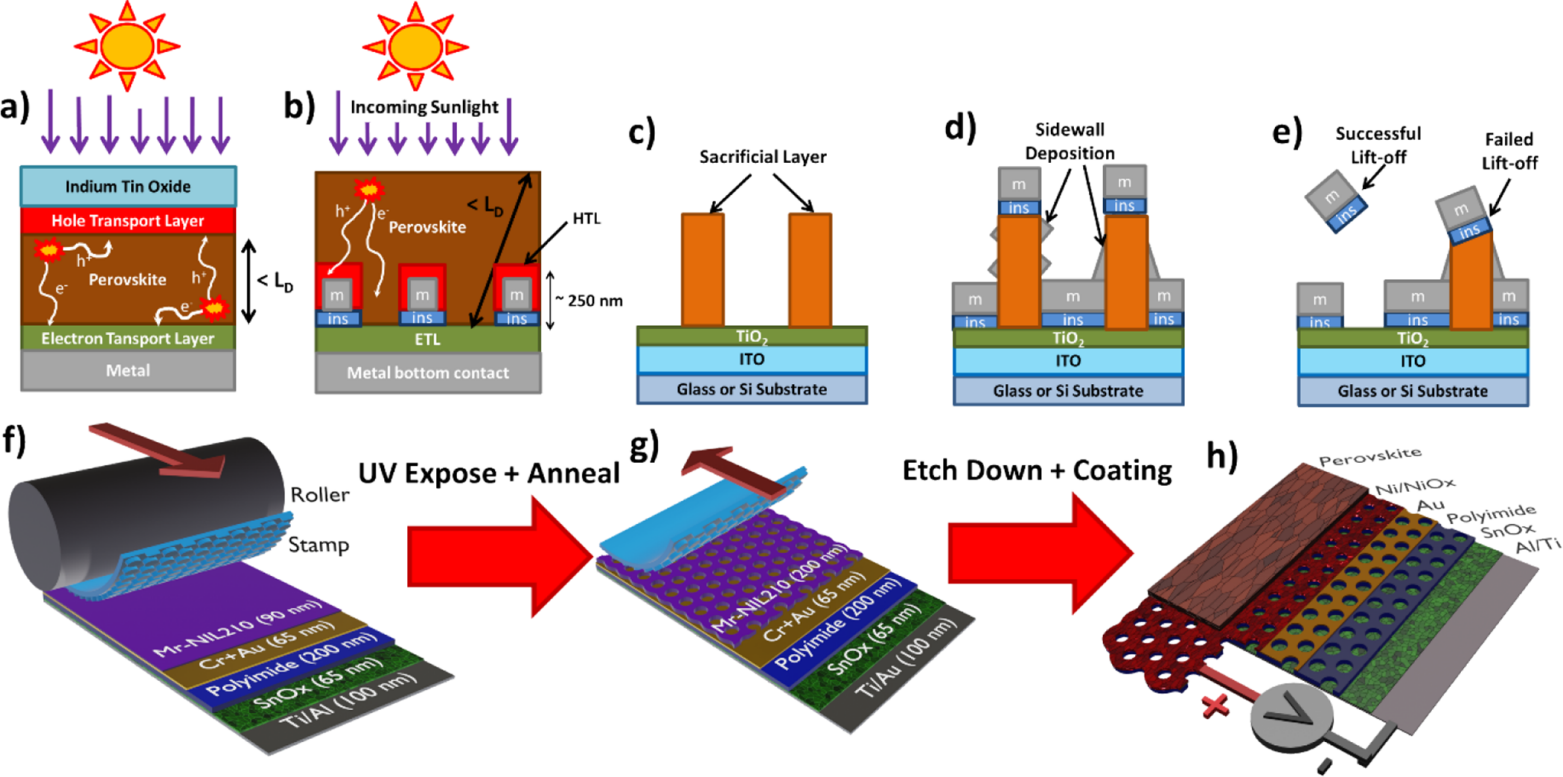
(a) Basic design of a conventional monolithic perovskite solar cell. (b) Basic design of quasi-interdigitated BC-PSC. (c–e) Process flow for the lift-off-based methods used thus far: (c) A sacrificial layer is patterned via a variety of methods. (d) The insulator and metal layers are deposited via evaporation. Note that some deposition on the sidewalls of the sacrificial layer will usually occur. (e) The sacrificial layers are dissolved by sonication in a solvent, thus removing the unwanted material. Excessive sidewall deposition will cause this step to fail. (f–h) Outline of the NIL process used in this work: (f) A stack of all the required materials is deposited with a UV curable resist on top. A stamp is then rolled onto the sample to imprint the pattern. (g) The resist is cured, and the stamp is peeled off. (h) The resist pattern is transferred to the target structure by plasma etching, and the perovskite is then deposited on top to complete the cell.

Perovskite solar cells (PSCs) are rapidly approaching widespread implementation due to their light weight,^1^ simple processing,^2^ and efficiency of up to 25.8%.^3^ The best performing devices currently use a monolithic structure (Figure 1a), where the active layer is sandwiched between an electron selective layer (ESL) and a hole selective layer (HSL), with conductive electrodes on either side to extract the charges. This architecture is simple to implement, but it is crucially limited by the fact that at least one of the electrodes must be made from a transparent conductor (TCO) to let light into the cell. TCOs are problematic due to their high cost,^4^ inflexibility,^5^ and reliance on rare and expensive materials such as indium. In addition, parasitic absorbance in the TCO layer can substantially reduce the efficiency of PSCs,^6^ and their relatively low conductivity becomes problematic when devices are scaled to large areas.^7^

A back-contact (BC) device is an alternative structure that works by using a series of interdigitated electrodes to collect all charges from just one side of the absorber, leaving the other side free to let light in unimpeded. It has already become popular in commercial silicon solar cells due to its ability to circumvent the need for TCOs, and there is now increased interest in exploiting this advantage in PSCs. To make a back-contact perovskite solar cell (BC-PSC), a quasi-interdigitated structure is used (Figure 1b), where the anode is patterned into a honeycomb structure and placed on top of the cathode with an insulating layer separating them.^8^ The diameter of the holes in the honeycomb electrode (*L*_E_) defines how far the charges must diffuse laterally before reaching their respective contacts, and device simulations have shown that *L*_E_ needs to be smaller than the charge diffusion length in the film to achieve internal quantum efficiencies, which match the monolithic structure.^9,10^ This is easy to achieve for silicon solar cells because the charge diffusion length (and hence the ideal *L*_E_ value) is in the tens of micrometers,^11^ which means that they can be made via simple contact photolithography techniques. However, the charge diffusion length in perovskite films is of the order 1 μm,^12^ which means that the ideal *L*_E_ value of a BC-PSC would be in the nanoscale. This presents a major, and so far unsolved, fabrication problem because achieving nanoscale electrodes on areas relevant to solar cells is difficult to do cost-effectively. Hence, most BC-PSCs reported so far have been limited to *L*_E_ values above 1 μm.

The first BC-PSCs were pioneered by Bach and co-workers, who used contact photolithography to pattern a quasi-interdigitated electrode with an *L*_E_ as low as 2.7 μm,^8^ and this was soon followed by Tainter *et al.* who showed that the efficiency of BC-PSCs rapidly increased as the *L*_E_ was reduced down to 1 μm.^13^ Other methods, such as direct laser scribing^14^ and aligned shadow masks,^15^ were tried but had low efficiency due to their *L*_E_ values in the tens of micrometers. Additionally, Lidzey and co-workers used angled evaporation on an embossed substrate to achieve efficient modules with an *L*_E_ of 1.6 μm and an estimated efficiency of 7.3%,^16^ but this was only achievable on an area of 10^–4^ cm^2^. To date, the most promising techniques for large-scale fabrication have involved “natural lithography” techniques, where a self-assembly process produces semi-ordered structures, which can then be used as a sacrificial template for depositing the structures via evaporation (this is known as a lift-off process). Prince *et al.* used the natural cracks that can be formed in a polymer film to produce quasi-interdigitated structures with an *L*_E_ of roughly 2 μm and a stabilized efficiency of 2.9%.^17^ Finally, Bach and co-workers set the world record for BC-PSCs by using microsphere lithography to achieve a stabilized efficiency of 8.6%,^18^ and this impressive result was attributed to their extremely low *L*_E_ value of 1.4 μm.

All the above works have shown that smaller *L*_E_ values are crucial to achieving high efficiency, and hence, there is great interest in pushing the *L*_E_ value into the nanoscale to make sure that it lies well below the *L*_D_ of perovskites. If the BC architecture were to also be implemented in the field of organic photovoltaic blends, where the free charge diffusion lengths are of order 100 nm,^19^ then the current *L*_E_ values would likely need to be further improved by an order of magnitude. Achieving sub-micrometer *L*_E_ values using lift-off-based approaches, which have been used in all BC-PSC reports thus far, is likely to be very difficult. A lift-off process works by making the patterns in a sacrificial layer (Figure 1c), depositing the desired layers via evaporation (Figure 1d), and then dissolving the sacrificial material to “lift off” the unwanted material (Figure 1e). However, this method becomes difficult to implement at very small feature sizes because unwanted deposition on the sidewalls of the sacrificial layer can inhibit the lift-off process. In their record-setting paper, Bach and co-workers report that these structures must be sonicated overnight to be completely lifted off, and even then they found that sacrificial spheres smaller than 2 μm could not be reliably removed. Our own findings in our lab corroborate this (see Figure S17), and this is a common problem in most other lift-off-based approaches, unless the sacrificial features have a high aspect ratio (it is generally assumed that they should be at least 3 times taller than they are wide).^20^

To solve the electrode width problem, we use UV nanoimprint lithography (NIL) in an etch-down process, thus circumventing the issues with lift-off entirely. NIL is a simple process where the desired features are copied from a pre-made master structure by simply stamping them into a UV-curable polymer (see Figure 1f,g). It can easily achieve feature sizes in the order of 20 nm^21^ and can be done using extremely low-cost equipment (in this paper, we use a UV torch and rolling pin). We use the NIL process in combination with reactive ion etching (RIE) to produce highly uniform electrodes with an *L*_E_ of 230 nm, 4 times smaller than the previous record. This allows us to produce BC-PSCs that are no longer limited by their charge diffusion length while also eliminating the need for carefully optimized self-assembly processes and the unreliable lift-off step. In addition, these techniques have great potential for scaling up to large areas, as commercial NIL systems already exist that can accommodate device areas on the square meter scale, while commercial RIE reactors are available that can work on a 500 mm × 500 mm area. Hence, there is great potential for incorporating these techniques in a high-throughput roll-to-plate process.^22,23^

## 2 Experimental Section

**Figure 2:**
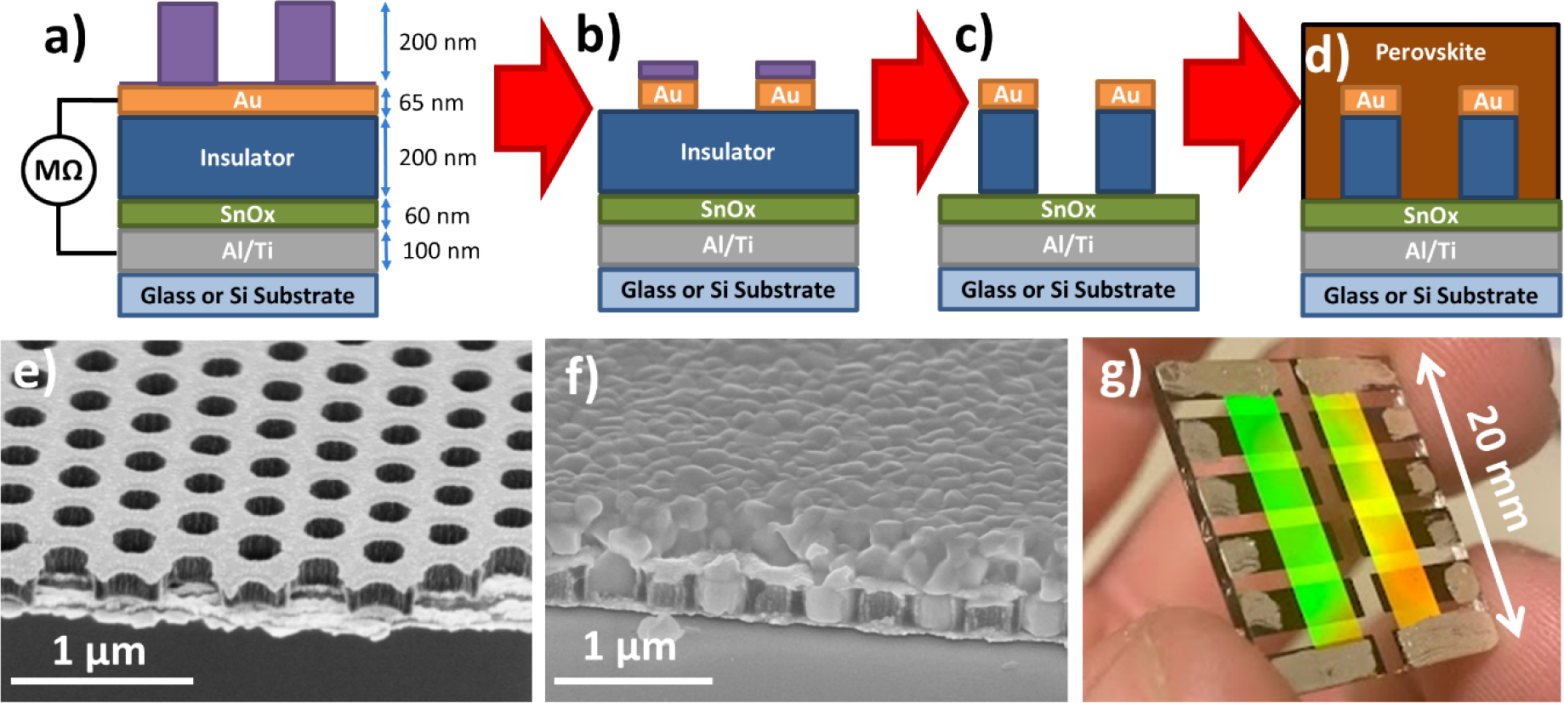
Process flow for making a BC-PSC via NIL: (a) First, a stack of Al/Ti, SnO_*x*_, PI, and Au is deposited on the substrate, and the desired nanostructures are imprinted in mrNIL-210 resist (purple) on top. (b) Argon plasma is used to drill holes in the gold, with the resist protecting the areas we want to keep. (c) The holes are further drilled through the insulating layer using oxygen plasma, also removing excess resist in the process. (d) The perovskite layer is deposited on top via spin-coating. (e) Cleaved cross section of a structure on panel (c) with an *L*_E_ of 230 nm. This device uses a silicon substrate for ease of imaging. The image is taken at a 45° angle. (f) Identical structure with perovskite deposited on top prior to cleaving. The total thickness of the perovskite layer in this image is approximately 600 nm, which results in a capping layer thickness of 335 nm. (g) Photograph of the device shown in panel (c), where 8 pixels are defined by the overlap between the titanium bottom electrodes (gray) and the honeycomb gold electrodes (iridescent lines). The iridescence in the honeycomb electrode arises from interference between the room light and the hole diameters of 230 nm.

The fabrication process described below and shown in Figure 2a–d is the exact procedure used in making the devices used for this publication and has been the subject of considerable optimization. For brevity, details such as the exact solution recipes and coating conditions have been moved to the Supporting Information. To assist other groups in reproducing this process, the Supporting Information also contains further information including common pitfalls in each step, viable material substitutions, alternative processing methods for low-resource labs, and the exact reasoning for each step in the process.

### 2.1 The Honeycomb Electrode

The basic structure for our BC-PSC is a honeycomb quasi-interdigitated design.^8^ This design (shown in Figure 1h) gives a high surface area for charge extraction, and the conductive grid gives good conduction while also being highly resistant to spot defects, since there are many possible routes for charge conduction. To make this via NIL, we first produce a capacitor-like structure consisting of planar metal electrodes separated by an insulating layer (Figure 2a). NIL is used to pattern a resist on top of this structure, which is then used as a mask to drill holes through to the bottom electrode using reactive ion etching as shown in Figure 2b,c. The perovskite layer is then deposited on top to complete the circuit as shown in Figure 2d. In this paper, we use an “n-i-p” structure, where the planar bottom electrode is the cathode, and the honeycomb top electrode will act as the anode.

The substrate was glass microscope slides, which were cut into a 20 mm × 20 mm square and then cleaned via the RCA cleaning method (detailed in the Supporting Information). A bottom electrode consisting of 60 nm aluminum followed by 40 nm titanium was deposited via electron beam evaporation, and the samples were cleaned again by sonication in isopropanol for 5 min followed by plasma ashing for 3 min in a commercial plasma asher. This specific choice of metal contact was crucial to achieving a working device, as most other metals caused the devices to be shorted (see the Supporting Information). A 60 nm-thick layer of tin oxide (SnO_*x*_) was then deposited to act as an electron transport layer (ETL) via spin-coating using the tin oxalate deposition route described by Cheng *et al*.^24^ This transport layer was chosen because we found it to be highly resistant to degradation during plasma processing, as detailed in Figure S3. Next, an insulating layer consisting of 200 nm polyimide (PI) was deposited by spin-coating a diluted solution of poly(pyromellitic dianhydride-*co*-4,4′-oxydianiline) and amic acid solution (Sigma-Aldrich) and annealing at 340 °C for 30 min. This insulating layer is crucial to ensuring a high shunt resistance in the final device, and any defects will cause catastrophic shorting. We found that organic insulating layers were more reliable for this task than inorganic dielectrics such as SiO_2_ or Al_2_O_3_ because they were less likely to form microcracks during handling or annealing, which could then act as shorting pathways, and PI was chosen specifically because of its ability to withstand heat treatments as high as 500 °C for short periods.^25^ This insulating layer should ideally be kept as thin as possible to minimize the amount of “dead space” in the device while also minimizing the vertical diffusion distance for electrons in the active layer. Finally, a cathode layer consisting of 65 nm gold with a 3 nm chromium adhesion layer (essential for preventing dewetting of the gold honeycomb, see Figure S4) was deposited on top of the PI via electron beam evaporation using a shadow mask to define pixel areas. The shadow mask patterns and the pixel layout are shown in Figure S2.

For the NIL step, the capacitor stack was plasma-ashed for 45 s to enhance wettability, and then a 90 nm layer of NIL resist (mr-NIL-210, Microresist Ltd.) was deposited on top via spin-coating. A flexible perfluoropolyether-based stamp (Fluorolink MD700, Acota Ltd.), containing a pillar array in a hexagonal grid pattern with a pillar height of 200 nm and a pillar width/separation of 230, 1000, or 2000 nm, was then gently pressed onto the sample by hand using a rolling pin (see the Supporting Information for fabrication details). Surface tension then holds the stamp in place, and the resist was cured by exposing with a 390 nm UV torch (30 mW/cm^2^) for 60 s. The stamp/sample combination was then placed on a hotplate at 100 °C, which further cured the resist and also caused the stamp to naturally delaminate from the sample (see Figure S6). The stamp was then washed with acetone and could be re-used at least 3 times before the imprint quality began to drop (see Figure S9).

The resist pattern was then transferred to the substrate via reactive etching. First, the gold layer was milled away using argon plasma, and the chromium adhesion layer was removed (along with the remaining resist) using a mixture of sulfur hexafluoride and oxygen plasma. Finally, holes were drilled into the PI layer using oxygen plasma, with the gold electrode acting as a hard mask. Silver paint is applied to the contact areas of the device to ensure a reliable connection during device testing, and the final result is shown in Figure 2g. At this point, the resistance between the anode and the cathode on each pixel is reliably in excess of 1 MΩ, and the sheet resistance of the gold honeycomb electrode is approximately 5 Ω/sq., which is superior to 15 Ω/sq. usually achieved by commercial indium tin oxide (ITO) substrates.^26^ The device was then completed by spin-coating a perovskite layer from a standard triple cation solution on top of the structure (see the Supporting Information). Cross-sectional scanning electron microscopy images of the final structure with and without the perovskite layer are shown in Figure 2e,f, and it can be clearly seen that the perovskite conforms to the structure and completely penetrates the holes down to the cathode.

### 2.2 The Hole Transport Layer

**Figure 3:**
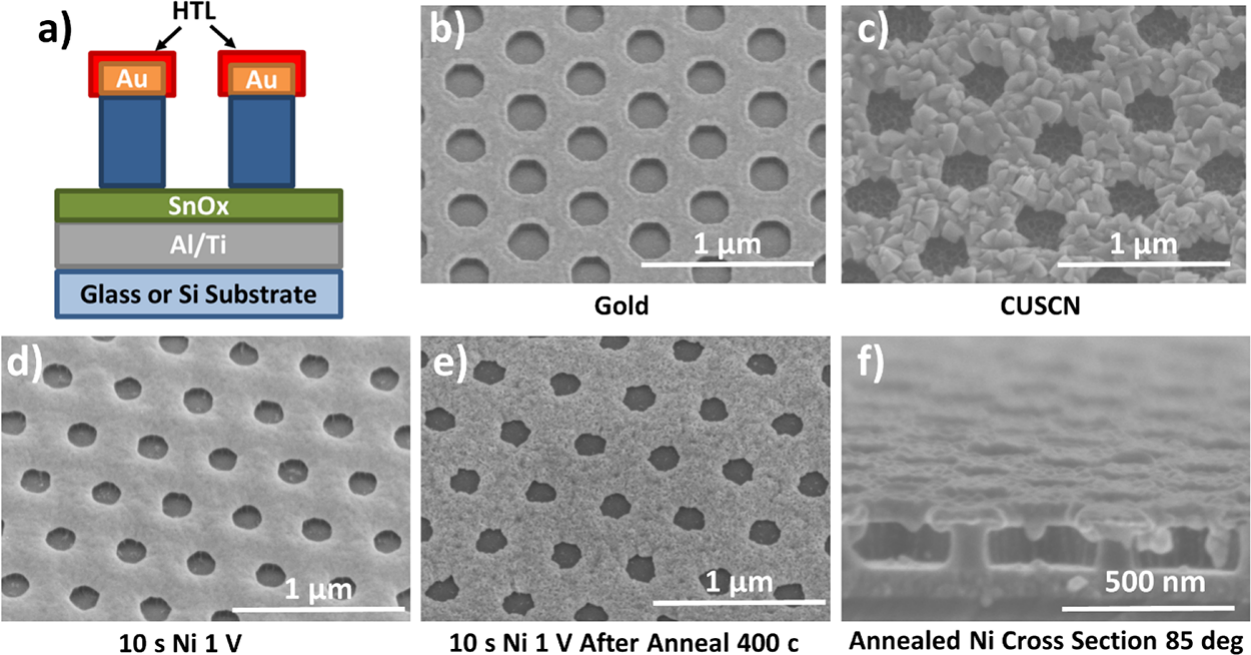
(a) Ideal target structure. We need a conformal coating of HTL around the gold honeycomb. (b) Angled (31°) SEM image of a bare gold BC-PSC with an *L*_E_ of 230 nm on a glass substrate. (c) Image of a similar device after CuSCN electrodeposition. (d) BC-PSC structure with Ni electroplated onto the gold honeycomb. (e) Ni device after annealing at 400 °C for 30 min. (f) Cleaved cross-sectional image (85°) of a Ni-based BC-PSC after annealing. Note that panel (f) was a sample based on a silicon substrate rather than a glass substrate because silicon can easily be cleaved into a clean edge for transverse SEM imaging.

Before the perovskite layer is deposited, a working device needs a hole transport layer (HTL) on the honeycomb top electrode, which must conformally cover the entire surface including the sidewalls. Without this layer, there will be large amounts of charge recombination at the perovskite/gold interface, which will result in poor efficiency. In addition, it must also be deposited exclusively on the gold electrode without causing any cross-contamination of SnO_*x*_ at the cathode(as shown in Figure 3a). This presents a major challenge because almost all of the techniques used to deposit HTLs, such as spin-coating,^27^ evaporation,^28^ chemical bath deposition,^29^ and sputtering,^30^ are generally not area-selective. That is, they will cover all surfaces equally, and thus, they are useless for this purpose. In this work, we use electrodeposition to overcome this issue because we can apply voltages to each electrode independently and thus ensure deposition only on the gold electrode, but this method is only viable for a select few materials. In addition to being selective, electrodeposition is desirable because it is an additive process that makes very efficient use of materials, requires low setup costs, and enables high throughput. Hence, it has frequently been touted as an excellent candidate for large-area solar cells.^31,32^

Copper(I) thioicyanate (CuSCN) and nickel oxide (NiO_*x*_) are popular HTLs, which have previously been reported to be deposited via electrodeposition,^33^ and so, these were chosen as the focus materials for this study. Following previous work by Ramachadran *et al*.,^34^ we found that CuSCN can be grown selectively on the gold electrode using a simple 2-electrode setup (see the Supporting Information for further details). Figure 3b,c shows angled SEM images of the BC-PSC structures before and after the optimized CuSCN electrodeposition. A densely packed film of CuSCN crystals with their distinctive triangular shape can be seen to form around the gold electrode, leaving the bottom electrode untouched. We note that this process does not work when using copper iodide (which is closely related to CuSCN) because it forms much larger crystals that do not conform to the electrode surface (see Figure S11).

While direct electrodeposition of NiO_*x*_ for PSCs has been previously reported using nitrate-based baths,^35^ we found that this route is not viable for BC-PSCs because the process relies on the creation of a cloud of OH^–^ ions around the electrode, which then causes the precipitation of Ni^2+^ to Ni(OH)_2_. This process is not localized enough to provide area selectivity on the length scales we desire, and hence, all our attempts to use this process resulted in significant cross-contamination (see Figure S12). Instead, NiO_*x*_ HTLs were formed by electrodepositing metallic nickel from a nickel chloride/sulfate bath and then annealing the whole structure at 400 °C for 30 min. As can be seen in Figure 3d, the electrodeposition process produces a smooth and conformal covering over the gold electrode without impacting the bottom electrode, and after annealing, it converts to polycrystalline NiO_*x*_ (as seen in Figure 3e). Despite annealing at temperatures greater than the official glass transition temperature (*T*_g_) of PI,^22^ we do not observe any substantial reflow in the features during this process, as can be seen in Figure 3f. This behavior has been previously seen in other organic materials such as SU-8^36^ and is usually attributed to a high degree of crosslinking, making the material resistant to reflow.

## 3 Results and Discussion

### 3.1 Effect of the Hole Transport Layers

**Figure 4:**
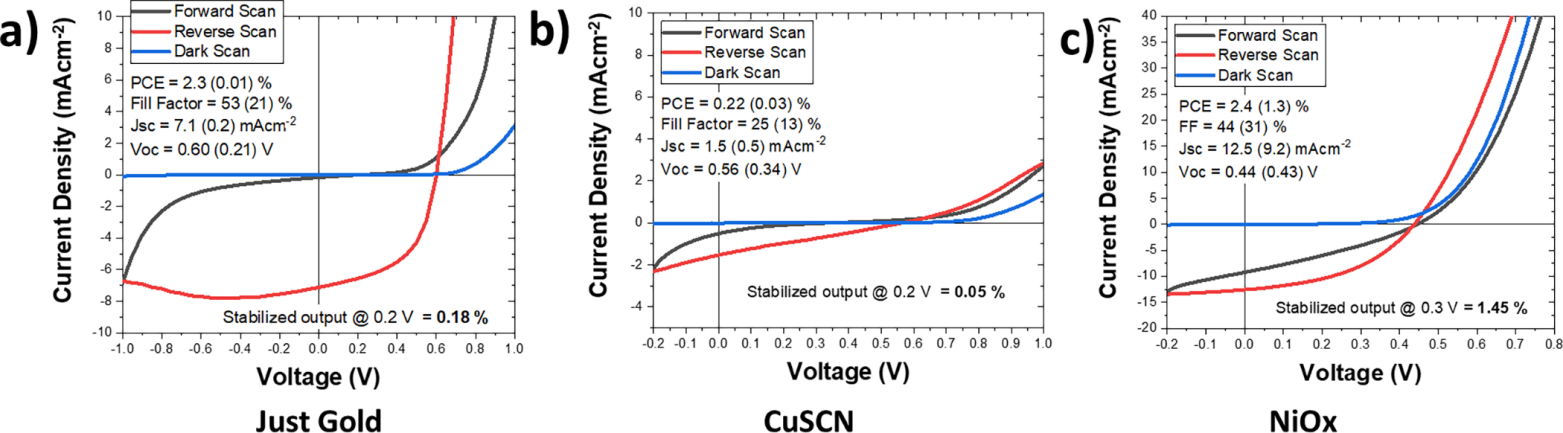
*J*–*V* curves for champion BC-PSCs with an *L*_E_ of 230 nm using different HTL materials on the gold top electrode. (a) Bare gold, (b) electrodeposited CuSCN, and (c) electrodeposited nickel followed by annealing at 400 °C for 30 min. The captions show the device parameters in reverse scan, with the forward scan results shown in brackets.

Using the above methods, we fabricated 3 batches of BC-PSCs with an *L*_E_ value of 230 nm. One batch had no HTL (bare gold), one batch had a CuSCN HTL, and the other had an annealed NiO_*x*_ HTL. The BC-PSCs were completed by spin-coating a perovskite layer using a triple-cation recipe. This recipe was chosen for its high reproducibility, and planar n-i-p cells with SnO_*x*_ and Spiro-OMeTad made using this recipe achieve power conversion efficiencies (PCEs) up to 16.5% in our laboratory (see Figure S3). The completed BC-PSCs were measured under AM1.5G light without encapsulation in a custom-made shadow mask setup with a 0.06 cm^2^ aperture. Figure 4 shows the *J*–*V* curves of the champion device from each batch, and it can be seen that the NiO_*x*_-based cells have by far the best performance with a stabilized power output (SPO) of 1.45%. On the other hand, the bare gold and CuSCN devices both show extremely large hysteresis and have low SPOs of 0.18 and 0.05%, respectively. The poor performance of the bare gold is not surprising because we would expect a large degree of charge recombination at the gold/perovskite interface, and there may even be electrically assisted chemical reactions contributing to the hysteresis. However, the poor performance of CuSCN is unexpected. The most likely explanation for this is that CuSCN is being partially dissolved by the perovskite precursor during the perovskite deposition. As shown in Figure S16, CuSCN is completely insoluble in both dimethyl formamide (DMF) and dimethyl sulfoxide (DMSO), but the addition of a small amount of perovskite precursor such as cesium iodide or formamidinium iodide enables rapid dissolution. Hence, the HTL is likely being washed away while the perovskite is being deposited, and this process could also catalyze a reaction between the perovskite and the gold to form gold iodide. This would explain why the PCE is even worse than a device with no HTL at all.

### 3.2 Effect of Electrode Width

**Figure 5:**
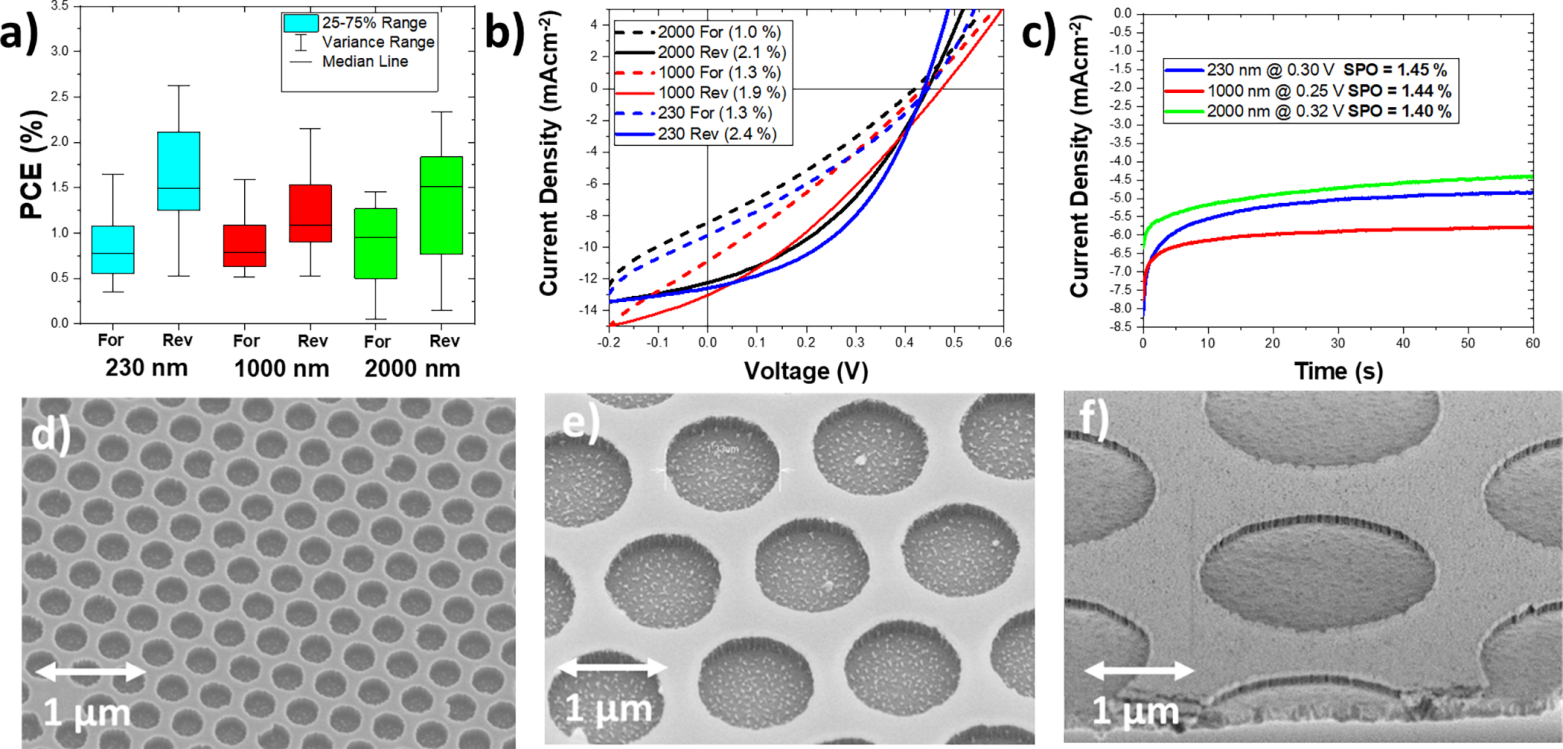
(a) Box and whisker plot of the forward and reverse scan efficiencies for NiO_*x*_-based BC-PSCs with different *L*_E_ values. (b) Champion *J*–*V* curves for each *L*_E_ value. The scan PCE values are shown in brackets in the figure legend. (c) Stabilized power output measurements for the champion device for each *L*_E_ value. The holding voltages and resultant PCE values are shown in the figure legend. (d–f) SEM images of 230, 1000, and 2000 nm BC-PSC structures at the same zoom level prior to nickel electrodeposition.

Through this process, we have created BC-PSCs with an *L*_E_ value of 230 nm, which is close to an order of magnitude smaller than the next smallest reported, but our SPO of 1.45% falls far below the 8.6% SPO record set by Bach and co-workers.^18^ Their work used a similar final device structure, but with an *L*_E_ of 1400 nm. Hence, it could be possible that reducing the feature size too much is actually detrimental to the performance of the device, perhaps due the decreased feature size making the electric field too weak near the surface of the perovskite layer. To investigate this, we fabricated new batches of devices all using annealed NiO_*x*_ as the HTL but with different *L*_E_ values of 230, 1000, and 2000 nm. Figure 5 shows the performance statistics, champion *J*–*V* curves, and stabilized power output curves of each batch. To our surprise, the average PCE and champion SPO values remain almost unchanged for all three *L*_E_ values, which would suggest that we have entered a regime where improving feature size no longer makes any difference.

This result is surprising because it goes against the predictions of drift-diffusion simulations previously reported in the literature.^9,10^ Although one might intuitively expect from the device geometry that an *L*_E_ value as large as 2 μm would be sufficient to collect all available charge (see Figure S1), these drift-diffusion models have shown that smaller *L*_E_ values than this are required for high performance. For instance, Ye *et al.* reported that a BC-PSC with a charge diffusion length of 1 μm and an *L*_E_ of 2 μm would only have an internal charge extraction efficiency of 70% and that *L*_E_ needs to be smaller than *L*_D_/2 (i.e., about 500 nm) to push this value above 90%. Following that logic, we would expect the short circuit current of the 230 nm cell to be at least 30% larger than the 2000 nm device, but our results show that they are essentially identical. A possible explanation for this could be that the charge diffusion length of the triple cation perovskite we used is substantially larger than 1 μm we had previously assumed. If the charge diffusion length was 4 μm or greater, then we could expect to be extracting 90% of the charges even with an *L*_E_ of 2 μm, at which point there would not be much benefit to reducing the *L*_E_ value further. This would be surprising but is not inconceivable, since there are some reports that mixed halide perovskites can achieve diffusion lengths up to 10 μm under the right processing conditions,^37^ and CH_3_NH_3_PbI_3_ single crystals have even shown *L*_D_ values exceeding 175 μm.^38^

### 3.3 Factors Affecting Efficiency

Given that the properties of the HTL appear to have a much larger impact on the PCE than the device geometry, we hypothesize that the reason for our low PCE when compared to Bach and co-workers is related to the quality of our NiO_*x*_ HTL and that future work should focus on improving this rather than on achieving small feature sizes. Other research groups have previously produced NiO_*x*_ HTLs by using a pure nickel electrode and carefully controlling the thermal oxidation to produce a thin surface oxide, with special care being taken to avoid oxidizing it all the way through. Our use of electroplated nickel on gold removes the risk of overoxidation, but it also creates the possibility that the gold could partially alloy with the nickel and thus contaminate the NiO_*x*_ surface layer. This would explain the low open circuit voltage (*V*_oc_) of only 0.4 V seen in our devices compared to the 1.0 V seen in other works. Gold is known to be a highly mobile material and has even been known to diffuse into the perovskite layer in monolithic cells, and so, it is also possible that gold contamination could be affecting the device performance. In addition to contaminating the perovskite, the gold could be diffusing into the PI insulator, thus reducing the device shunt resistance. Another possibility is that the gold could be coating the SnO_2_ layer on the bottom electrode. However, our observations noted in Figure S4 would suggest that large-scale reflow of the gold onto SnO_2_ is unlikely. Future work will focus on modifying our fabrication techniques to enable a pure nickel top electrode and will also look into alternative area-selective deposition techniques for HTLs and ETLs.

We note that, given the likely impact of the thermal NiO_*x*_ layer on the performance of the cell, it would be ideal to have some normal planar devices incorporating this layer as an HTL. However, this proved to be impossible to achieve because the transparency of the Ni/NiO_*x*_ layer was too low to let light into the device.

### 3.4 Recyclable BC-PSCs

**Figure 6:**
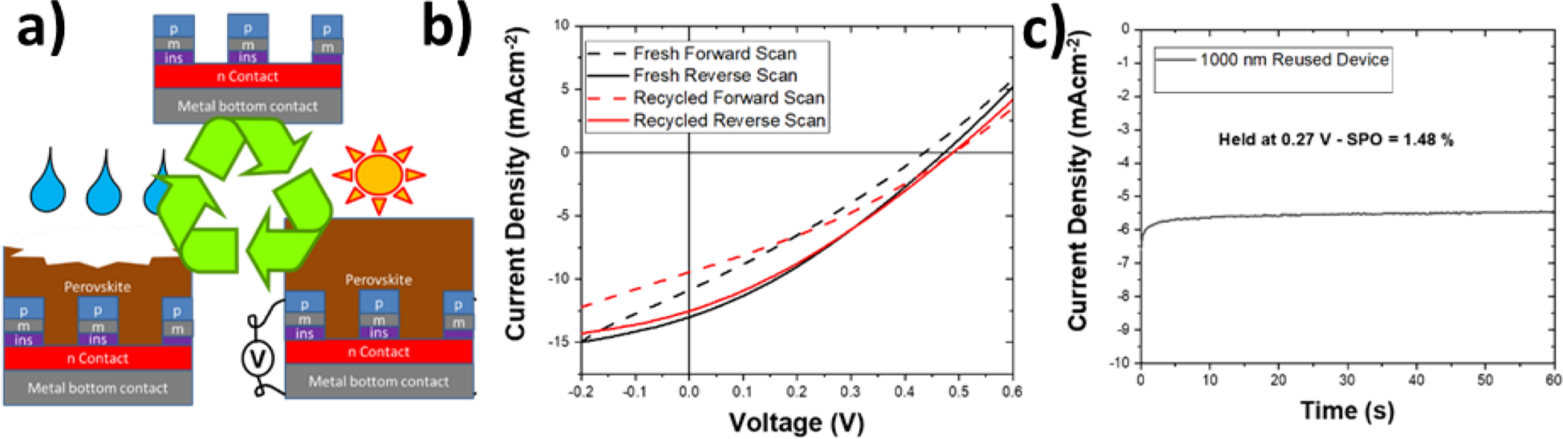
(a) Procedure for recycling a BC-PSC. A completed cell is washed with DMSO, leaving a clean BC-PSC structure. The fresh perovskite can then be redeposited on top to make a new cell. (b) *J*–*V* curve and (c) SPO measurement of a NiO_*x*_-based, 1000 nm *L*_E_ BC-PSC when fresh and *J*–*V* curve of the same device after it had been aged for 1 week and then recycled.

A final potential advantage of BC-PSCs that we want to explore is their potential for easy recycling. In a planar PSC, if the active layer becomes degraded, then the entire device must be dismantled back down to the original substrate and then remade from the start. Here, we show that a degraded BC-PSC can be far more easily recycled simply by washing off the degraded perovskite and then re-depositing it from a fresh solution as shown in Figure 6a (also see Video S1). To do this, we took an old BC-PSC and removed the perovskite by dipping it in a solution of dimethyl sulfoxide (DMSO), which rapidly dissolves the perovskite but does nothing to any other layer. The clean BC-PSC substrate was then regenerated by annealing at 400 °C for 30 min. Once the samples had cooled, a fresh perovskite layer was deposited via spin-coating and the solar cells were remeasured. Figure 6 shows the *J*–*V* curve and SPO of our champion 1000 nm BC-PSC device when brand new and compares it to the same cell after it was aged in air without encapsulation for 1 week and then regenerated with a fresh perovskite layer. It can be seen that the resulting *J*–*V* curves are almost identical, and the champion SPO of 1.48% is even slightly better than the original best of 1.44%. This shows that BC-PSCs have great potential to be easily re-used and recycled on large scales.

## 4 Conclusions

In summary, we have demonstrated a top-down route to fabricating honeycomb quasi-interdigitated electrodes that eliminates the problematic lift-off processes used in previous reports. This capability will have many applications for novel optoelectronic devices such as electrically pumped photonic crystal lasers, vertical organic field-effect transistors, and in-plane organic LEDs. Its compatibility with NIL enables us to achieve an *L*_E_ value of 230 nm, which is close to an order of magnitude smaller than the previous record *L*_E_ of 1400 nm,^18^ and its simplicity and ease of use will make BC-PSC production more accessible to the wider research community. However, we find that the efficiency of our BC-PSCs remains almost unchanged for *L*_E_ values between 230 and 2000 nm, leading us to conclude that BC-PSCs have now reached the point where device geometry is no longer their limiting factor. Instead, we find that the properties of the HTL are far more critical to device performance, and so, future work should focus on finding ways to deposit higher quality charge transport layers onto the honeycomb contact of the BC-PSC. Finally, we show that BC-PSCs can be quickly and easily recycled with no noticeable reduction in their performance. This feature would be of great benefit to ensuring a zero-waste life cycle for commercial perovskite solar cells.

## Supporting Information Available

Video showing the process of removing perovskite from a degraded BC-PSC for regeneration (MOV)
Detailed fabrication notes, discussion of optimal honeycomb geometry, alternative processes that were tried, challenges for scaling up to large areas, dark *J*–*V* curves, and surface profilometry data (PDF)

## Supplementary Material

an3c02493_si_001.mov
