# Supplementary material for: Nanoimprint Lithography as a Route to Nanoscale Back-Contact Perovskite Solar Cells
Source: ACS Appl Nano Mater. 2023 Aug 16;6(16):14940–7. doi: 10.1021/acsanm.3c02493 (PMC10463217; doi:10.1021/acsanm.3c02493)
Supplement: Supplementary file 2 — an3c02493_si_002.pdf [file an3c02493_si_002.pdf]

Nanoimprint Lithography as a Route to Nanoscale Back-Contact Perovskite Solar Cells - Supporting Information

Jonathon Harwell[1], Ifor D.W. Samuel[1]*

1. University of St Andrews, School of Physics and Astronomy, North Haugh, St Andrews, KY16 9SS, United Kingdom

*Email: jrh8@st-andrews.ac.uk

Definition of L_E and Optimal Honeycomb Design

The key parameter of note in a BC-PSC is L_{Max}, which is the maximum distance a charge will have to diffuse before reaching its respective contacts. This value, and the point at which it occurs, will be different for electrons and holes, as shown in figure S1. L_{Max} is determined by the thickness of the perovskite layer (which gives the vertical component) and the electrode width L_E (which determines the lateral component). **For consistency, we shall define L_E as the diameter of the holes in the honeycomb lattice, which in the case of this study is either 230 nm, 1000 nm, or 2000 nm** (Note that this is not the same thing as minimum dimension or feature size). The maximum lateral diffusion length for a positive charge is when a photon is absorbed in the middle of one of the circular gaps in the honeycomb, in which case it is simply half the diameter of the gap, or $L_E/2$.

For electrons, the maximum lateral diffusion length is when a charge is created at one of the "tri-points" in the honeycomb, where it is equidistant from three holes in the grid. We would expect that the optimal design would have close to equal diffusion lengths for both charges, and so we designed the centre-to-centre spacing of the holes in our honeycomb to be 1.732*L_E, while the row-to-row spacing is 1.5*L_E. This means that the maximal lateral diffusion length will be $L_E/2$ – exactly the same as for holes.

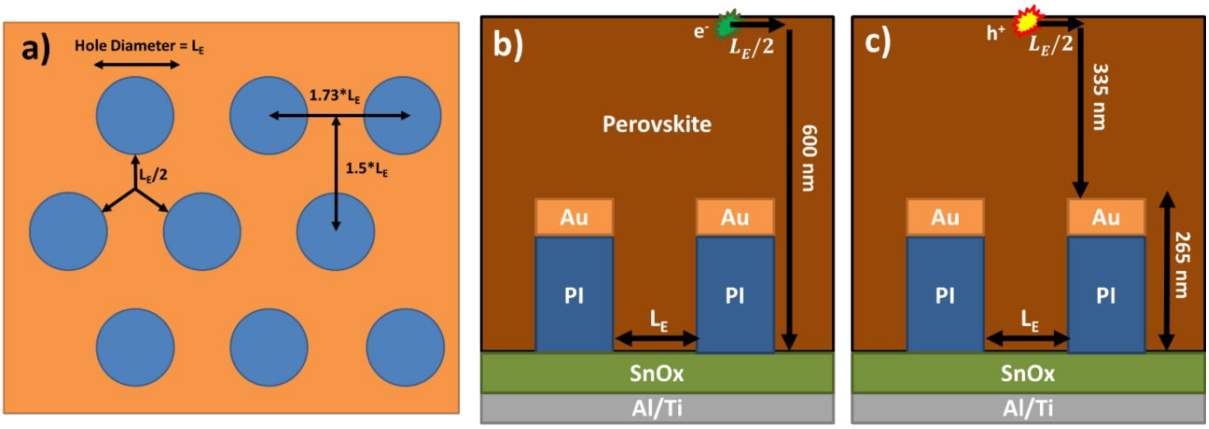

Figure S1 – a) A top-down view of the honeycomb electrode design, where gold represents the conducting grid and blue represents the drilled holes down to the cathode. b)The maximum vertical and lateral diffusion distance for electrons. c) The maximum lateral and vertical diffusion length for holes.

For electrons, the vertical component of L_{Max} is simply the thickness of the perovskite layer (we assume 600 nm), while for holes we subtract the thickness of the cathode (65 nm) and the insulating layer (200 nm), giving a vertical component of 335 nm. The absolute value of L_{max} can now be calculated using Pythagoras theorem, and the results for electrons and holes are shown on table S1. Note that the insulating layer should be as thin as possible so as to minimise the volume of "dead space" which the insulator takes up, while also minimising the vertical diffusion distance of electrons for a given capping layer thickness.

L_E Value	Lateral L_{Max}	Holes Vertical L_{Max}	Electrons Vertical L_{Max}	L_{Max} Holes	L_{Max} Electrons
230 nm	115 nm	335 nm	600 nm	354	611
1000 nm	500 nm	335 nm	600 nm	601	781
2000 nm	1000 nm	335 nm	600 nm	1054	1166

Table S1 – Maximum linear diffusion distances for electrons and holes in BC-PSCs with different L_E values.

If we assume a charge diffusion length of 1000 nm, even an L_E value of 2000 nm will put most charges within the diffusion length of their contact. However, previously published drift-diffusion simulations predict that this is not sufficient for complete charge collection, and an L_E value of 2000 nm would only result in an internal quantum efficiency of 70 %, while an LE value of 500 nm would be required to exceed 90 % internal quantum efficiency[1].

Detailed Fabrication Process

Pixel Design and Evaporation Masks

Solar cells for this work were fabricated on 20 mm x 20 mm square substrates with an electrode configuration as shown on figure S2. Cathode strips 2.5 mm wide and 9 mm long are evaporated first, and the interlayers are deposited via spin coating. The anodes are then deposited as two 4mm wide strips, with 8 pixels defined by the overlap points. The overall pixel area is 0.1 cm2, but a shadow mask with an area 0.6 cm2 is used to define the illumination during measurement.

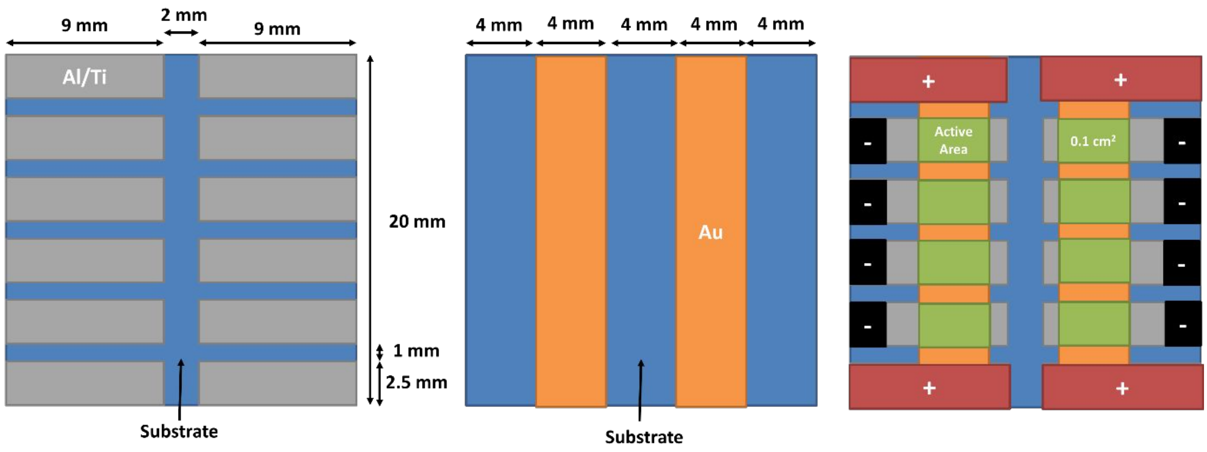

Figure S2 – (a) The Mask design for the first metal deposition (cathode). (b) The mask design for the second metal deposition (anode). (c) The final pixel layout, where the active pixel areas are highlighted in green, the anode contact areas are red, and the cathode contact points are black.

Substrates

The BC-PSCs made here use glass substrates cut from standard float glass microscope slides using a diamond wheel cutter. Samples for imaging cross sections via scanning electron microscopy were made on (100) monocrystalline silicon wafers, which were cut to shape by scribing and then cleaving against a razor blade.

Notes

-Flexible substrates can also be used, but are much harder to work with and we were unable to get consistent enough results for publication here. Polyimide substrates are in theory ideal for this purpose because of their high heat resistance, and their poor transparency is not an issue for BC-PSCs.

Cleaning

These devices are extremely vulnerable to short circuits caused by dust contamination, so **cleanliness is vital to reliable fabrication**. All fabrication was performed in a class 1000 cleanroom, with as much work as possible being done inside a laminar flow hood at class 100 dust rating.

The cut substrates are first scrubbed by hand with a lint-free cloth wetted with isopropanol to remove any strongly adhered grease or particulates, and then immersed in a solution of 1 part ammonia (30 %, Sigma Aldrich), 1 part hydrogen peroxide (30 %, sigma Aldrich), and 5 parts deionised (DI) water for 30 minutes at 70 ^{o}C (This is widely known as the RCA method). After this they are rinsed with DI water, then sonicated sequentially in DI water, acetone, then isopropanol for 5 minutes each. Finally the samples are cleaned in an oxygen plasma asher or UV-Ozone cleaner for at least 3 minutes.

Notes

- **No cleaning surfactants are used**, as we found that they are prone to leaving residues even after repeated rinsing in deionised water.

-The hand scrubbing is important because we found that sonication is excellent at removing fine particles, but heavy soiling or strongly adhered particles are not effectively removed. The scrubbing

cloth removes these very effectively, and any fine particles left by the cloth can be easily lifted off during sonication.

-We found that devices made without using the RCA step still work, but approximately 20 % of the pixels were shorted.

-Note that hydrogen peroxide waste should not be disposed of in the same container as organic waste as this can cause an explosion

Bottom Electrode (Cathode)

The optimised electrode was deposited through a shadow mask via electron beam evaporation in an angstrom nexdep electron beam evaporator at a pressure of 1x10^{-6} mbar. **A 3 nm Cr adhesion layer (not essential) was deposited at 0.1 nm/s, then a 60 nm Al layer was deposited at 0.5 nm/s. Finally a 40 nm Ti was deposited at 0.5 nm/s**. All layers were done on a rotating stage without breaking vacuum between layers. Using this configuration, the bottom electrode had a sheet resistance of ~ 0.4 Ω/sq. After deposition, the samples are briefly sonicated in isopropanol to remove any dust from the evaporation chamber, dried with an N2 gun, then treated in a plasma asher for at least 45 s to make a hydrophilic surface.

Notes

ITO or FTO can easily be used as an alternative bottom electrode, but the potential for TCO-free devices is one of the key selling points of BC-PSCs, so we focussed on using a metal electrode for the bottom contact.

We found that the choice of metal for the bottom electrode is crucial for ensuring a working device. Devices using bottom electrodes made from coinage metals such as silver, gold, platinum, or copper would always be catastrophically shorted in 100 % of their pixels. We hypothesise that this is caused by the diffusion of metal ions into the insulating layer. We have no way to test this conclusively, but metal ion diffusion is known to be a common problem in semiconductor devices[2] so it is not unreasonable to expect it here. More reactive metals such as nickel, chromium, aluminium, and titanium did not cause shorting, which may be because their reactivity and higher hardness prevents diffusion, or that any ions which do diffuse are quickly oxidised into poorly conducting oxides.

Nickel and chromium will both form oxide layers which are unsuitable for the "n-i-p" device structure pursued in this paper, so they cannot be used here (the possibility of of "p-i-n" devices are briefly discussed later). Pure aluminium was also found to be unsuitable because it forms a highly insulating Al_2O_3 layer on its surface, which resulted in extremely high series resistances in the final devices. Titanium forms a (poor quality) n-type TiO_2 layer oxide on its surface, which makes it the best available option for BC-PSCs, but pure Ti films have poor conductivity, so aluminium was deposited prior to Ti deposition to act as a supporting electrode. Note **only aluminium can be used as the supporting electrode** – we found that supporting electrodes made from silver or copper would still cause catastrophic shorting even with a 200 nm thick Ti layer on top of them. If Al deposition is not available, pure Ti electrodes do work, but will have increased series resistance (it's probably best just to use ITO at that point).

Thick metal layers (particularly Nickel) have a tendency to flake off during cleaning with sonication. The 3 nm chromium adhesion layer helps to prevent this.

Electron Transport Layer

We used the tin (II) oxalate method first shown by cheng et al[3], where 0.75 M (154 mg) of tin (II) oxalate (sigma Aldrich – product 402761) was added to 1 ml of 30 % wt hydrogen peroxide solution (Sigma Aldrich – product 1.08597). After a few seconds, rapid bubbling is observed and the solution becomes extremely hot, before stabilising to a clear and strongly acidic solution. This solution is filtered in a 0.45 micron filter and then spin coated in air at 4000 RPM for 30 s. They are then annealed at 180 °C in air for at least 10 minutes. A pale blue, dust-free film is seen on the samples after spin coating, which rapidly turns colourless when placed on a hotplate.

Notes

The presence of solvent vapours (such as acetone, dimethylformamide, or chlorobenzene) during the spin coating seems to encourage the formation of aggregates, which results in very poor quality films. To prevent this, the spin coater must be lined with fresh foil prior to spin coating the SnOx solution so that residual solvent vapours from previous samples don't ruin the film.

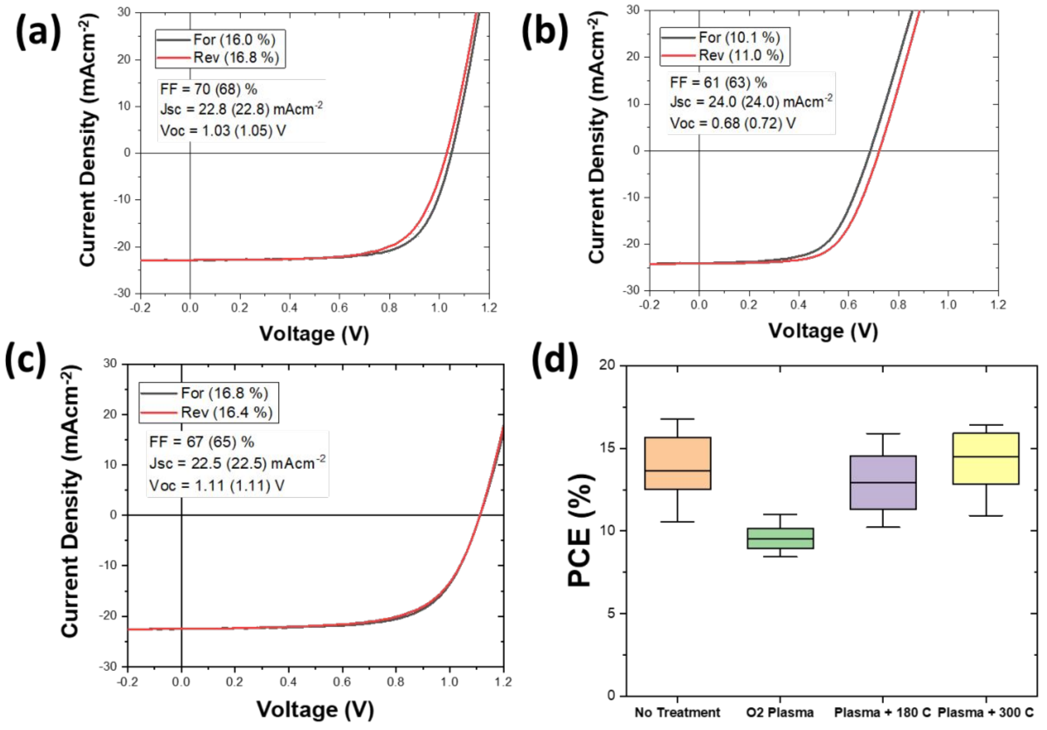

Figure S3 – J-V curves for planar n-i-p PSCs with SnOx as the electron transport layer, CsMAFA as the active layer, and Spiro-OMetad as the hole transport layer with 50 nm Au as the top contact. a) control device, b) device where the SnOx layer is treated with O_2 plasma for 3 minutes prior to perovskite deposition, c) the same as b) except that the SnOx is "healed" with a 300 C post annealing treatment after the plasma exposure. d) Box and whisker plots for the device batches.

The native oxide layer on the Ti electrode was not sufficient to provide good charge transport properties, even when annealed at 500 °C to convert it to the anatase phase (oxidised Ti metal has frequently been found to give poor performance in solar cells when used alone).[4] Organic ETLs cannot be used because they will be destroyed by the oxygen plasma during the etch down step, and any inorganic ETLs must be able to maintain good properties even after plasma exposure. We used SnOx as a transport layer because many groups have shown that it can survive or is even improved by exposure to O_2 plasma[5].

To test the O_2 resistance of the SnO_x films, we fabricated normal planar n-i-p perovskite solar cells of the structure ITO/SnOx/perov/Spiro/Au, and the SnOx films were subjected to O_2 plasma treatment for 60 s, followed by annealing at various temperatures prior to perovskite deposition. The perovskite layer was the triple cation recipe described later, and the hole transport layer was made following Ossila's optimised spiro-ometad recipe[6]. As shown on figure S3, the control devices (with no plasma exposure) achieve an efficiency of ~16 %, which drops substantially if the film is exposed to O_2 plasma with no heat treatment. However we found that a post annealing step at 300 ^oC can completely recover the performance to match the untreated control. This shows that this SnOx layer will be ideal for our etch down process because we do not need to worry about plasma damage during the etching.

We note that the efficiency of the devices achieved here are rather low when compared to literature results, where efficiencies above 18 % are frequently achieved with the same structure. The reason for this is unclear. Despite the low PCE, this experiment still performs its purpose by showing that plasma damage can be recovered by thermal annealing. Other groups attempting to reproduce our results may find that they get better back contact device efficiencies than reported here as a result of this.

The Insulating Layer

For the insulating layer, a solution of Poly(pyromellitic dianhydride-co-4,4'-oxydianiline), amic acid (12.8 % wt – sigma Aldrich 575798-250ML) was diluted in a 1:2.5 volume ratio with dimethyl formamide (DMF) solvent. This solution was filtered in a 0.45 micron filter then spin coated at 4000 RPM at 4000 RPM for at least 90 seconds. The samples are then placed on a hotplate at 340 ^oC for 30 minutes to complete the imidisation reaction and make a 200 nm thick polyimide (PI) film.

Notes

Note that an extended spin coating time is required to fully evaporate the DMF solvent, which has extremely low volatility at room temperature. Films are a pale yellow colour coming off the spin coater, and it is crucial that they have no visible dust particles. A rapid colour change is observed when the samples are placed on the hot plate. No ramping of temperature is needed – they can be placed directly on the hotplate. After the annealing, samples must be allowed to cool to room temperature before handling.

PI was chosen as an insulator because it has a high thermal resistance while still having the flexibility and reliability of organic insulators. We found that inorganic insulating layers (e.g. SiO_2 or Al_2O_3) were very unreliable whether they were deposited via spin coating (as a sol-gel) or via evaporation, and hence we were unable to achieve working devices with them (all of them shorted at some stage of the fabrication). We hypothesise that this is due to the formation of microcracks in the film during annealing or handling, which create shorting pathways between the electrodes.

Other organic insulating layers were also explored. Poly(methyl methacrylate) showed excellent and reliable insulating properties with thicknesses as low as 50 nm, but its low glass transition temperature and high solubility in various solvents made it useless for our devices. The epoxy-based photoresist SU-8 (TF6001 – Acota materials) can also be used by spin coating a film at least 200 nm thick and then flood exposing it with a UV torch to cross-link it. SU-8 has similar reliability to PI films, but it becomes brittle and discoloured at temperatures above 300 ^oC, which means that most devices became shorted during the final annealing step to convert Ni to NiOx.

The Top Electrode (Cathode)

The top electrode was deposited through a shadow mask via electron beam evaporation in an Edwards electron beam evaporator with a rotating sample holder at a pressure below $8x10^{-6}$ mbar. A 3 nm chromium adhesion layer was deposited at 0.05 nm/s, followed by a 65 nm gold layer at 0.1 nm/s without breaking vacuum between the layers (the exact rate doesn't matter much). After deposition, the samples were plasma ashed for 45 s to make their surface hydrophilic for the nanoimprint lithography (NIL) step.

Notes

The gold layer must be deposited from a tungsten crucible – NOT a graphite one. Gold layers deposited from a graphite crucible were found to interact with the nanoimprint resist deposited in the next layer, causing premature cross linking where the resist touches the gold and ruining the device. We believe that this is due to carbon contamination entering the gold film, which causes a chemical reaction with the compounds in the resist.

The chromium layer is kept as thin as possible to make it easy to etch through, but it must be at least 2.5 nm thick. In samples with chromium layers thinner than this, we observed that the gold layer could dewet during the later annealing steps (see figure S4). This could be observed as a sudden rise in the sheet resistance of the gold layer.

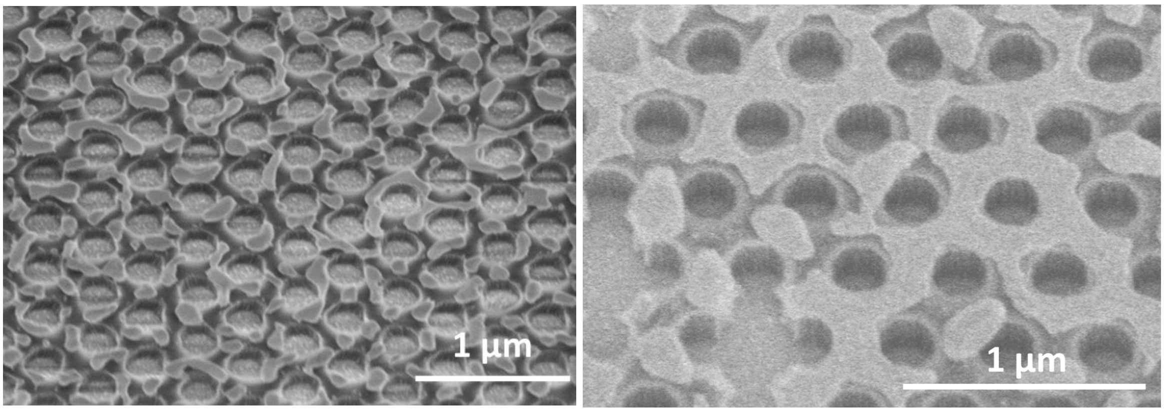

Figure S4 - What happens if a Cr adhesion layer is omitted. The device works fine initially, but when the sample is annealed above 100 C the gold honeycomb will dewet into discontinuous islands which do not conduct. Note that even in this extreme condition where the gold is able to reflow freely without an adhesion layer or nickel coating there does not appear to be significant movement of gold into the holes. Hence there is unlikely to be large scale covering of the SnO_2 with gold during the annealing step.

The effectiveness of the insulating layer can be tested at this point. Measuring the resistance between the top and bottom electrodes should reliably achieve resistances exceeding 1 MΩ, while the sheet resistances of each electrode should be less than 3 Ω/sq

Nanoimprint Lithography – Master Structures

Master structures for the NIL process came from two different sources. The 230 nm period master was the "MLH230/200/460-30x30" standard silicon mold, purchased from Scivax (rough cost of 4000 USD). It is a 30 mm x 30 mm silicon sample with a nano-hole array in a hexagonal array with a hole diameter of 230 nm and a lattice period of 460 nm. The hole depth was 200 nm. The 1000 nm and 2000 nm master structures were made using custom quartz/chrome photomasks purchased from JD Photodata inc. Hole arrays made from 100 nm chromium with the design shown on **figure S1** were supplied by JD Photodata (rough cost of 500 USD), and we then etched the masks via reactive ion

etching to increase the hole depth to 200 nm. Prior to use, the master structures were treated with a non-stick layer by placing them in a vacuum chamber overnight with a few drops of 1H,1H,2H,2H-Perfluorooctyl-trichlorosilane (Sigma Aldrich 448931-10G) to create a self-assembled monolayer.

Nanoimprint Lithography – Stamp Fabrication

The master structures were then used as templates for stamps made from Fluorolink MD700 (purchased from Acota – Approx 1500 USD/kg). A UV-curable resin was made by adding 3 wt % darocur 1173 photoinitiator to the MD700, which was stirred overnight and then degassed in a vacuum chamber. This mixed resin can be stored in an amber bottle indefinitely. To make the stamp, a master structure was placed on a level surface, and then the resin was dropped onto the master and spread to cover the whole structure (Approx. 1 ml covers a 30 x 30 mm area). The liquid resin was then exposed with a 390 nm UV torch with an intensity of approximately 30 mW/cm^2 for 30 s. This will solidify most of the resin, but a thin layer of uncured resin will remain on the surface because the cross-linking reaction is inhibited by contact with air. To complete the curing, a glass cover slip is carefully floated on top of the resin to exclude as much air as possible, and then is left to be illuminated by the torch for approx. 5 minutes. After curing, the cover slip is peeled off and the stamp/master combo is rinsed with acetone to remove any remaining uncured resist. The MD700 stamp can then be carefully separated from the master using a scalpel blade.

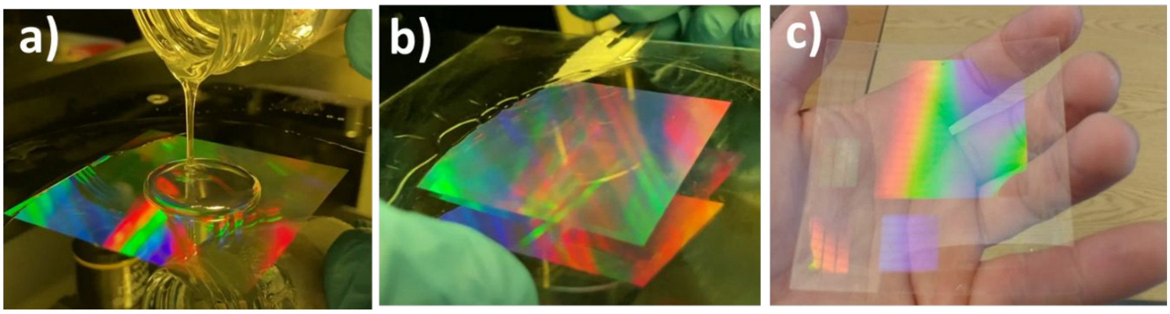

Figure S5 - Stamp Fabrication - a) MD700 is poured onto a master structure, covered with a glass coverslip, and cured under UV. b) The cured stamp material is peeled away from the master. c) An example of large-area NIL, where a sample of PET which has been imprinted with mr-NIL nanostructures using a large-area MD700 stamp.

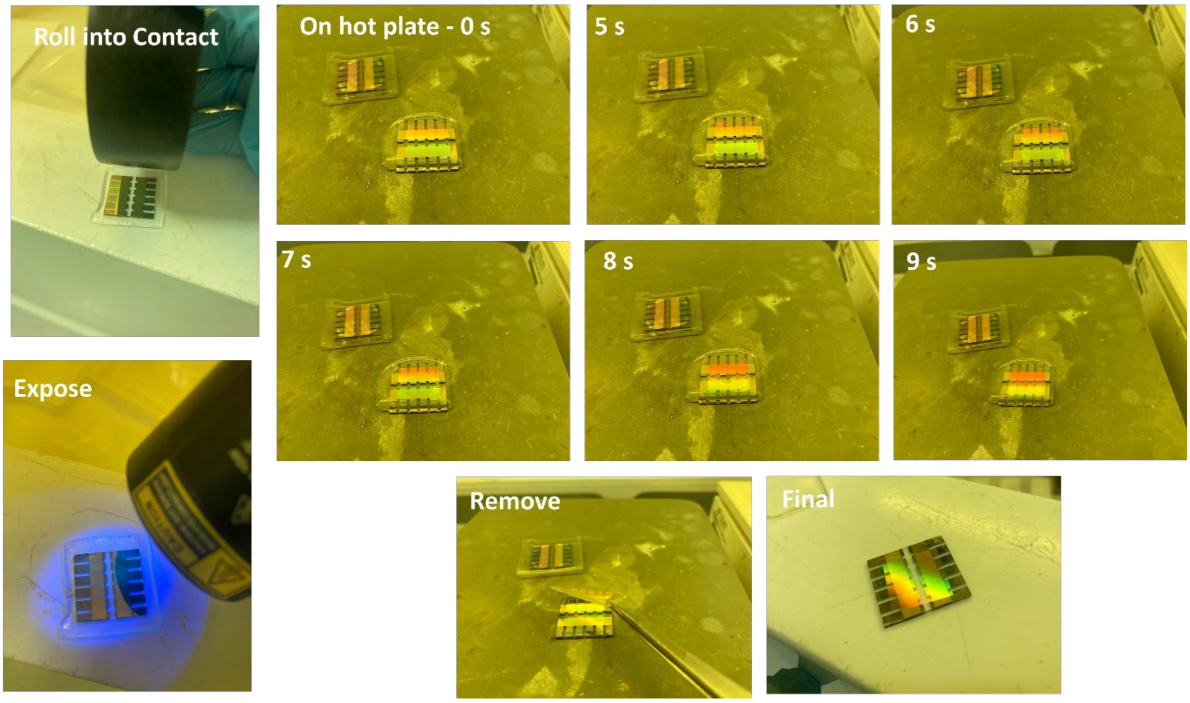

Figure S6 – Imprinting on a sample – the flexible stamp is gently placed on top of the resist-coated sample, and then pressed into conformal contact with a roller. It is then cured with a uv torch, and placed on a hotplate. The stamp will spontaneously separate from the sample, leaving the imprinted resist behind.

Nanoimprint Lithography - Imprinting

Once the stamps have been fabricated, a layer of imprint resist is spin coated onto the BC-PSC samples. For this purpose, we use mr-NIL 210 – 200 nm resist (micro resist technology) diluted in a 1:1 ratio with mr-t-1078 thinner solution (micro resist technology). This solution is spin coated at 2000 RPM for 30 s in air to make a 90 nm thick film. After spinning, a stamp is carefully placed on top of the sample, and is then forced into contact with the sample by gentle pressure with a rolling pin, taking care not to allow the formation of bubbles. Surface tension ensures that the stamp stays in contact with the sample. Once complete contact is achieved, the resist is cured by exposure with a UV torch (390 nm, 30 mW/cm2) for at least 60 s. The stamp/sample combo is then placed onto a hotplate at 100 ^{o}C. The heat from the hotplate completes the resist crosslinking reaction and also causes the MD700 stamp to curl away from the hotplate, thus causing it to spontaneously separate from the sample, leaving the sample with the desired pattern imprinted onto it. The used stamp is then rinsed with acetone and dried for reuse.

Notes on Nanoimprint Lithography

MD700 is perfluoropolyether (PFPE)polymer with acrylate termination groups. These acrylate groups cross-link with each other during the curing process, which is what makes the film go solid. PFPE is chosen because it is soft and flexible, thus ensuring perfect contact with the target for NIL, while the high fluorine contact minimises adhesion between the stamp and the imprint resist. Minimum adhesion is essential to ensuring that the stamp can separate from the sample without peeling off the resist with it.

The MD700 resin will go touch hard after only a few seconds of curing, but it is important to cure the film as long as possible (several minutes) because any un-crosslinked acrylate groups will provide anchors for adhesion between the NIL resist and the stamp. This will prevent the stamp and the

resist from cleanly separating. If the stamp does not spontaneously separate from the sample during annealing, it is usually because the stamp was not cured for long enough.

The mr-nil resist is still liquid after spin coating, and only goes solid upon UV exposure. When the stamp comes into contact, surface tension will cause it to reflow to fill in gaps in the stamp. The film thickness of 90 nm is chosen to be slightly less than half the feature height of the stamp (200 nm) so that the resist completely fills the gaps but leaves behind minimal residual layer.

As an alternative to mr-nil resist, urethane arcrylate resins used for modelling (e.g. limino clear UV resin - amazon product number B087373N5F) work almost as well as commercial NIL resists – the resin can be thinned down by diluting approx. 1:10 in cyclopentanone and then used in the same way as mr-nil210. It leaves a small amount of residue during dry etching, but otherwise operates equivalently.

Alternative stamp materials were tried but were not as reliable as MD700 – Ormostamp is a hard stamp material produced by micro resist technology, but due to its high rigidity it would rarely get a perfect contact with the sample, meaning that patterns could not be reliably replicated in the mr-NIL resist. Poly dimethyl siloxane (PDMS) based stamps were also tried, but were significantly more time consuming to produce, could not achieve as high resolution as MD700, and also could not be separated from the resist as easily.

Etching the patterns

The patterned mr-NIL210 was then used as a resist to transfer the pattern to the BC-PSC structure. This was done in a home-built reactive ion etcher (RIE). First, the residual layer was removed using oxygen plasma for 5 s (RF power: 20 W, DC bias: 300 V, O_2 flowrate: 200 sccm, pressure: $1x10^{-1}$ mbar, resist etch rate: 100 nm/min).

Next, the 65 nm gold layer was etched using argon plasma for 100 s (RF power: 100 W, DC bias: 740 V, Ar flowrate: 500 sccm, pressure: $3x10^{-2}$ mbar, resist etch rate: 70 nm/min, gold etch rate: 50 nm/min).

Next, the chromium adhesion layer was removed using an SF_6 and O_2 mixture for 30 s (RF power: 20 W, DC bias: 150 V, O_2 flowrate: 200 sccm, SF_6 flowrate: 100 sccm, pressure: $4x10^{-2}$ mbar, resist etch rate: 200 nm/min). The volatile etch product is chromyl fluoride CrO_2F_2. The remaining resist is also completely removed in this step, but the gold is not significantly affected.

Finally, holes are drilled into the PI layer with oxygen plasma for 3 minutes. (RF power: 20 W, DC bias: 300 V, O_2 flowrate: 200 sccm, pressure: $1x10^{-1}$ mbar, PI etch rate: 100 nm/min). The gold is not substantially etched in this step. The etch time is deliberately more than necessary to remove the 200 nm of PI, as this ensures a residue-free bottom contact.

Notes

These etch rates and gas parameters are specific to the instrument used in our lab, and will need to be re-optimised for other instruments

If SF_6 is not available, chlorine can be used instead to etch chromium. A mixture of CHF_3 or CF_4 with O_2 will also work but not as efficiently.

The gold etching mechanism using argon is purely sputter based. Our RIE was not capable of etching any other metal in this way because the ion energy was not large enough. Other groups with more

powerful systems may have success in etching other metals – if a substantial thickness of nickel could be etched then it would eliminate the need for the electrodeposition step below.

If a full RIE setup is not available, a plasma asher (to etch the resist and the PI) in combination with a wet etch (for the gold and the Cr) can be used instead, albeit with reduced performance. Note that a UV ozone cleaner is not sufficient to etch organics, and the plasma asher will have substantially less directionality than a full RIE system. First, the residual resist layer must be removed with a 45 s O_2 treatment in a plasma asher. The gold can then be etched in a mixture of potassium iodide and iodine in water in a 1:1:100 weight ratio. This etches gold at approximately 50 nm per minute. The chromium can then be removed using a commercial chromium etching reagent (Sigma Aldrich 651826-500ML). Finally the PI layer can be etched with 15 minutes O2 treatment in the plasma asher. Using this method, 230 nm feature sizes can be achieved with difficulty. Note we were only able to achieve this using a gold electrode. Wet etching on other metals such as copper was much less controllable due to the formation of oxide layers with different etch rates to the bulk metal. Hence the minimum resolution was in the micron range. The common etchants for metals such as nickel are also extremely acidic, and would frequently cause peeling of the masking resist.

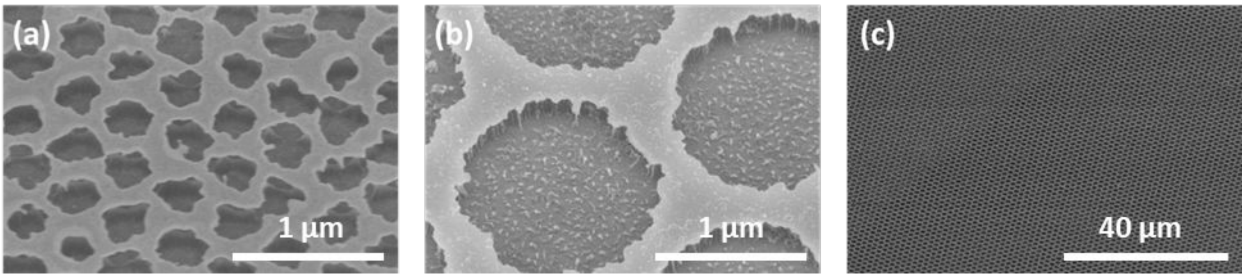

Figure S7 – BC-PSC Honeycombs made without an RIE – the gold is wet etched with $KI+I_2$, and the PI is etched with an O_2 plasma asher. (a) shows 230 nm features, (b) and (c) show 1000 nm features at different zooms. Note that the holes are much more irregular sizes, and there is substantial undercutting of the PI layer in the 230 nm device.

After etching the metal top contact, the sheet resistance is observed to increase by a factor of 2-3 from the original value. This is expected given that we are removing roughly 50 % of the metal, and defects or overetching will increase the resistance further. If the resistance does not increase by a factor of at least 2, it is usually a sign that gold has not been completely etched through, and the argon milling time needs to be increased.

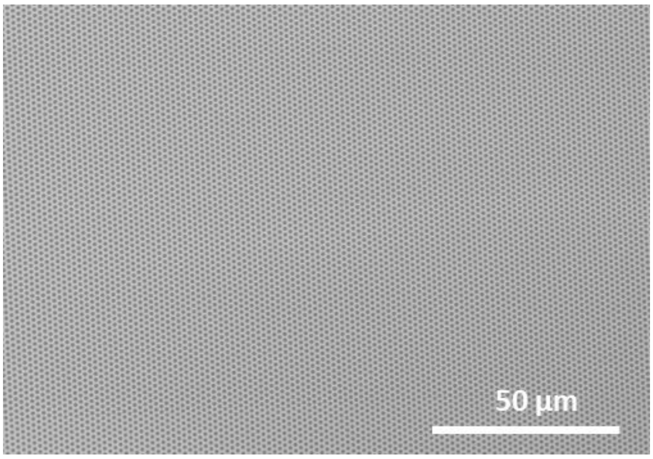

Figure S8 - Uniformity of NIL - This shows a 1 micron BC-PSC zoomed out to a roughly 0.2 mm x 0.2 mm area. No defects are visible in the entire scan area

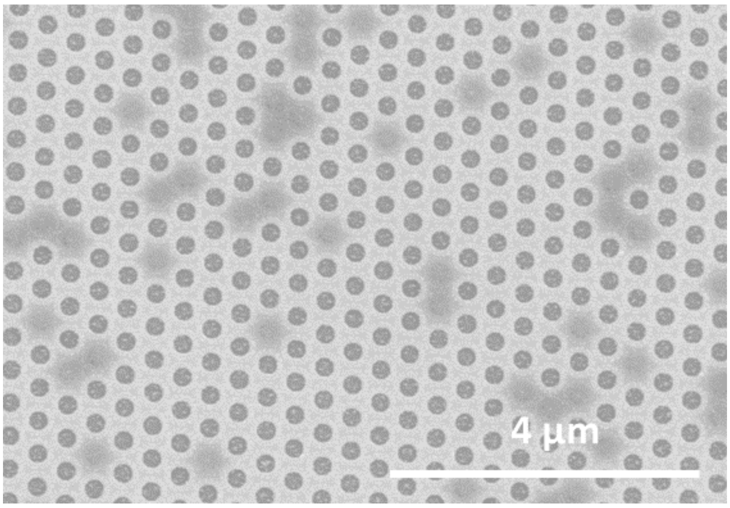

Figure S9 – The effect of stamp reuse – The above shows a 230 nm BC-PSC structure made using a stamp which had been re-used 6 times. The NIL still works, but progressively more and more of the holes fail to appear. This does not completely kill the device, but will result in reduced PCE.

Electrodepositing the Transport Layers

Nickel Oxide

The gold electrodes were coated with metallic nickel using a neutral $NiSO_4$ bath in a simple 2-electrode setup as shown on figure S10. The gold honeycomb electrode was used as the cathode, while the anode for electrodeposition was a piece of pure nickel. The Ti/SnOx electrode in the solar cell was left floating with no electrical contact – no current could flow through it so no deposition occured. The samples were held at a potential difference of 0.5 V between the nickel anode and the gold cathode for a period of 30 s. Longer deposition times resulted in thicker nickel films, but increased the likelihood of a short circuit between the gold electrode and the Ti/SnOx electrode. At 60 s deposition time we found that roughly 30 % of the pixels are shorted.

The bath composition is as follows:

40 ml DI water

9 g $NiSO_4$ (sigma Aldrich)

1.2 g $NiCl_2$ (sigma Aldrich)

Stir until clear and then filter through a 0.45 um PTFE filter.

The deposition chemistry is as follows:

Cathode: $Ni^{2+}_{(aq)} + 2e^- \rightarrow Ni_{(s)}$

Anode: $Ni_{(s)} \rightarrow Ni^{2+}_{(aq)} + 2e^-$

Once the ni has been deposited, the samples are rinsed with DI water, dried, and then placed on a hotplate at 400 C for 1 hr to convert to NiOx. Samples should be heated up and cooled down relatively slowly (max 50 ^{o}C/min) or the thermal shock will cause them to shatter.

Note that commercial nickel plating solutions are not suitable for this purpose because they usually contain brighteners which contaminate the nickel, resulting in impure NiO_x layer. In addition, they tend to be acidic, which results in the dissolution of the SnO_x layer.

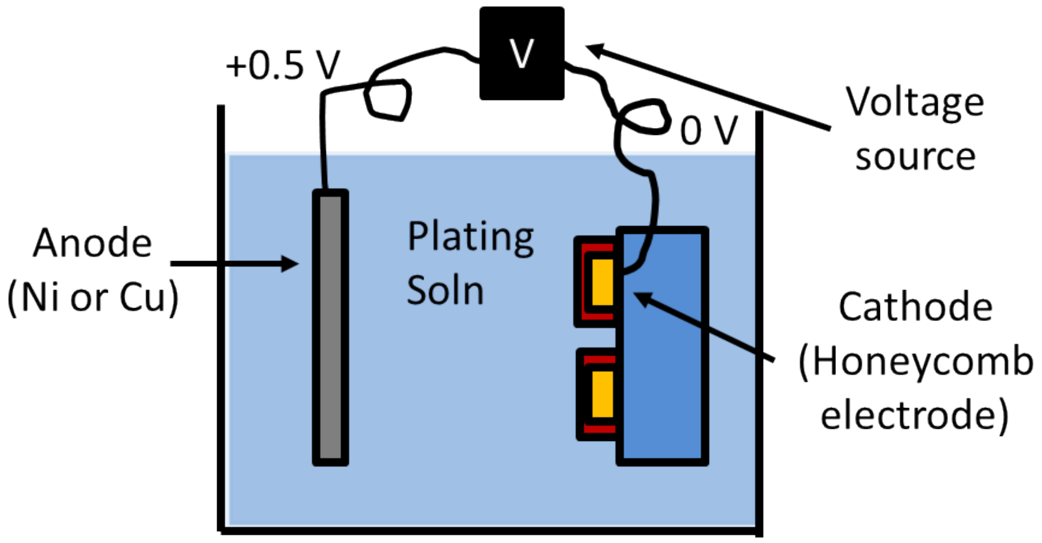

Figure S10 – Simple schematic of the electrodeposition setup used in this work.

CuSCN Electrodeposition

CuSCN is deposited in a 2 electrode cell in a modified version of the procedure set out by Ramachran et al.[7] Similar to the Ni procedure, the gold honeycomb electrode is used as the cathode for electrodeposition while the SnOx electrode is left floating. The anode for electrodeposition is a plate of pure copper metal (NOT platinum as used in the literature). The plating solution is as follows:

12 mM (3.0 mg/ml) Copper Sulfate pentahydrate (Sigma Aldrich)

12 mM (1.2 mg/ml) Postassium thiocyanate (Sigma Aldrich)

12 mM (3.5 mg/ml) Ethylene diamine tetraacetic acid (EDTA) (Sigma Aldrich)

The purpose of the EDTA is to stabilise the solution and prevent the solution precipitating out prematurely.

For plating, a 0.1 V potential difference is applied for 60 s. The following reaction occurs at the anode and the cathode:

Cathode: $Cu^{2+}_{(aq)} + e^- + SCN^-_{(aq)} \rightarrow CuSCN_{(s)}$

Anode: $Cu^0_{(s)} + SCN^-_{(aq)} \rightarrow CuSCN_{(s)} + e^-$

After deposition, samples were rinsed with DI water, blow dried, and then annealed on a hot plate at 90 ^{o}C in a glovebox prior to perovskite deposition.

Note that CuSCN forms on both electrodes here, and the Cu electrode must be periodically cleaned with a dilute ammonia solution to remove the grown CuSCN. The above reaction requires almost no driving force, and hence requires a very low voltage (it will even occur slowly at 0V potential difference). A copper anode is essential, because otherwise the only balancing reaction which can occur at the anode is the water splitting reaction, which requires high voltage.

We also tried this deposition method with CuI, where the KSCN in the plating solution is replaced with potassium iodide. We found that CuI grows much larger crystals than CuSCN, but they were not

conformal to the electrode, and hence they could not be used (see figure S11).

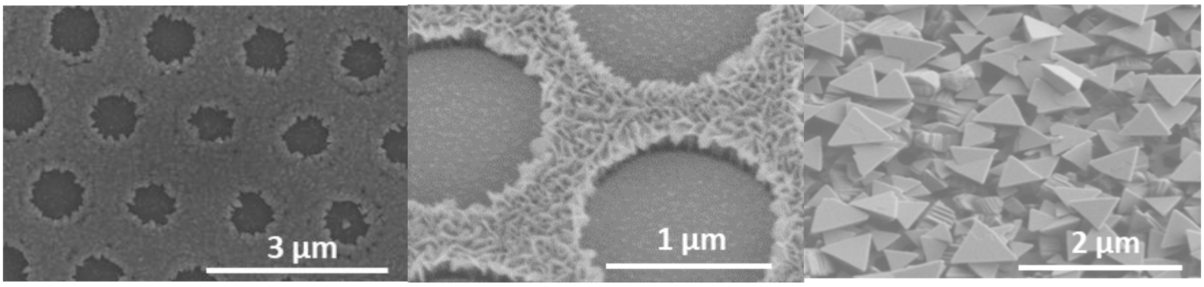

Figure S11 – Electrodeposited (left) CuSCN, (middle) CuISCN, and (right) CuI

Other Routes

We also explored using nitrate-based plating solutions as routes to directly deposit NiO_x or SnO_x layers, but they rely on the following reaction at the cathode to work:

Cathode: $NO_3^- + 2e^- + H_2O \rightarrow NO_2^- + 2OH^-$

Cathode step 2 (for NiOx): $2OH^-_{(aq)} + Ni^{2+}_{(aq)} \rightarrow Ni(OH)_{2(s)} \rightarrow NiO_{(s)} + H_2O$

The reaction works by creating a cloud of OH^- ions around the electrode, which causes precipitation of the metal ions into metal hydroxides, which can then be decomposed into the target oxide. This cloud of OH^- ions is not sufficiently localised to cause selective deposition in the way we want, and hence will always result in cross-contamination as shown below.

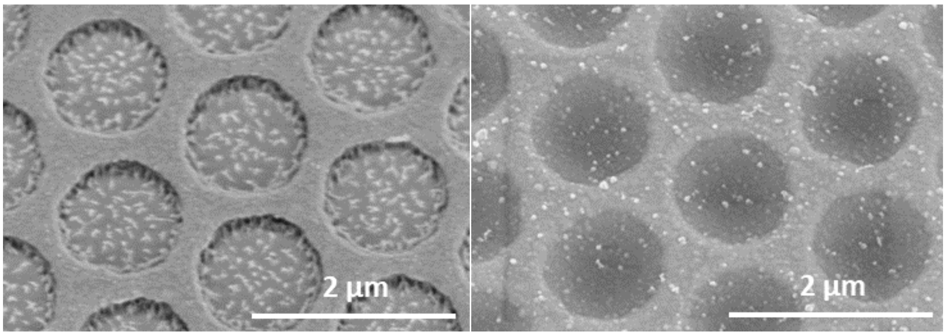

Figure S12 – 1 micron grid before and after cathodic NiOx edep from a 0.1 M $NiNO_3$ solution

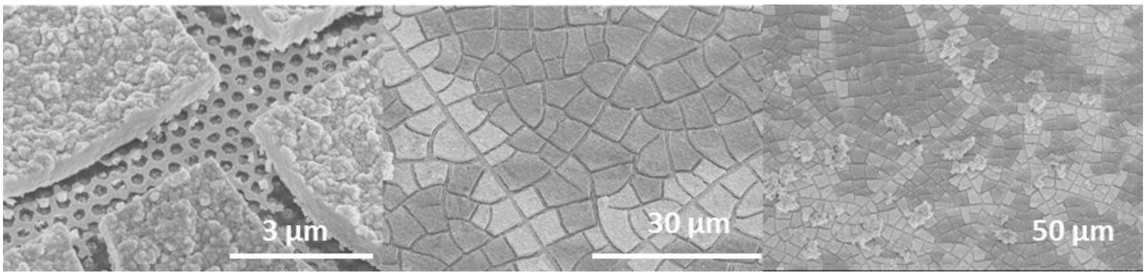

Figure S13 – 230 nm grid after attempts to electrodeposit SnO_2 from a $SnCl_2/HNO_3$ solution

We also tried using anodic deposition to deposit materials such as NiOOH from nickel acetate, or SnO_2 from an $SnCl_2/H_2O_2$ solution, but none of these appeared to deposit anything.

Contact Pads

Prior to deposition of the perovskite film, the contact points for the devices were coated with a layer of conductive silver paste and then dried at 100 ^oC for 20 minutes. This makes it easy to make a good

contact with the devices during testing. It does not appear to affect the perovskite deposition.

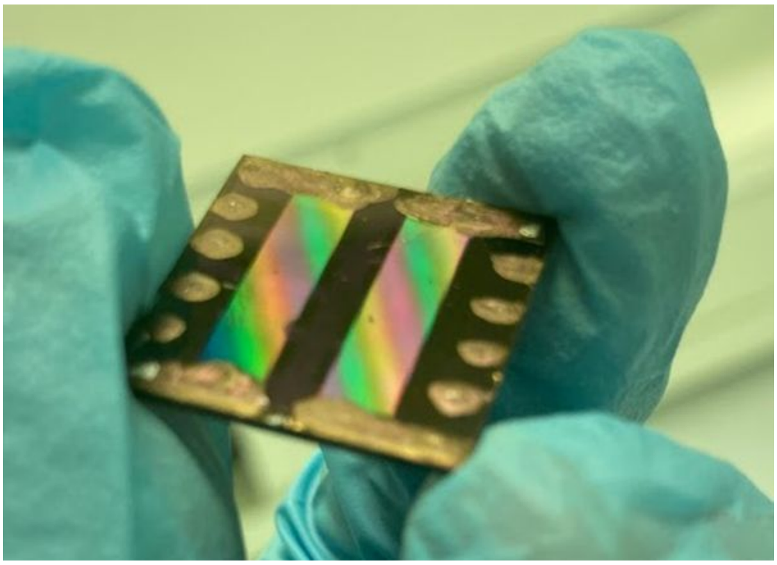

Figure S14 - A finished BC-PSC with the perovskite added. Note that the interference effects from the electrode are still visible, and the painted contacts are free to be accessed during testing

Perovskite Deposition

For perovskite deposition, we used the triple cation recipe which has been extensively used in previous reports. Briefly, a 1.5 M (390 mg/ml) stock solution of cesium iodide (sigma Aldrich) in DMSO was made, and then 507 mg PbI_2 (perovskite grade, TCI) and 73.4 mg $PbBr_2$ (perovskite grade, TCI)were weighed out into a vial. This powder was then dissolved in 1 ml of solvent (DMF:DMSO in a 4:1 ratio) and then 57 µl of the CsI stock solution was added. In a second vial, 22.4 mg methylammonium bromide (perovskite grade, TCI), and 172 mg formamidinium hydroiodide (perovskite grade, TCI) were weighed out into a second vial. The contents of the first vial was then added to the second vial, and the mix was completely dissolved with vigorous shaking.

The samples to be coated are placed in a spin coater in a nitrogen filled glovebox, and 100 µl of solution is dropped onto the sample, being careful to make sure that all the pixel areas are completely covered. The sample is then spun at 1000 RPM for 10 s and then at 6000 RPM for 30 s. 10 s into the second step, an antisolvent consisting of 600 µl of ethyl acetate is rapidly dripped onto the sample. The sample is observed to turn a translucent brown colour. Once the spinning finishes, the sample is placed on a hotplate at 100 ^{o}C for 1 hour. Upon contact with the hotplate, the sample immediately becomes much darker. Final films should be dark brown and mirror-smooth.

We note that the exact spin coating conditions seem to vary significantly between labs, even when in gloveboxes with ostensibly the same O_2 and water content. We hypothesise that residual solvent vapours in the glovebox can significantly affect the spin coating process, and so we recommend changing the lining of the spin coater regularly, and minimising the amount of solvent stored in the glovebox as much as possible.

Device Testing

Devices are tested in air without encapsulation. A custom sample is used, with a shadow mask area of 0.06 cm^2. Contact to the pixels is made through pins touching the silvered contact pads. The shadow mask is held 1 mm away from the sample surface to ensure that the mask does not scratch the devices. Device area is defined by the illumination area of the shadow mask.

Samples are illuminated with a Sciencetech AM1.5 G solar simulator, and tested using an Ossila sourcemeter. Stabilised power output is measured by visually determining the maximum power point of the best device, and then holding the device at that voltage for 60 seconds while measuring the current output. The SPO is defined as the power output in the final data point of this measurement.

Samples are stored in air under dark conditions.

Cross-sectional SEM

All SEM measurements were done in a Hitachi S4800 electron microscope.

To do cross-sectional SEM measurements, identical samples are made using silicon (100) substrates instead of glass. We use substrates with a thermal oxide layer of at least 300 nm, as this prevents conduction occurring through the Si substrate. Note that the conductive nature of the Si substrate can change the etch rates during RIE substantially (up to 2x faster etching!), so this must be taken into account during fabrication. Completed samples are then cleaved by making a small cut in the silicon and then pressing it onto a razor blade. This causes a crack to propagate between the crystal planes, resulting in a very clean edge ideal for SEM measurements. The cleaved sample is then placed on a specialised holder for imaging at oblique angles. If Si substrates are unavailable, it is also possible to get 45 degree images on a glass substrate by simply scoring the back of the sample with a diamond scribe and then snapping it, but the cut is never as clean as a Si substrate. This makes it much more difficult to find a sharp edge for imaging.

Calculating Electrode Sheet Resistance

Electrode sheet resistance measured using a two-probe method by measuring the resistance across the full length of one of the cathode rails in the device structure shown on fig S2c. An upper limit for the sheet resistance is obtained by assuming the strip length to be 15 mm long by 4 mm wide. Shunt resistance is obtained by measuring the resistance between an anode and cathode with no perovskite absorber present.

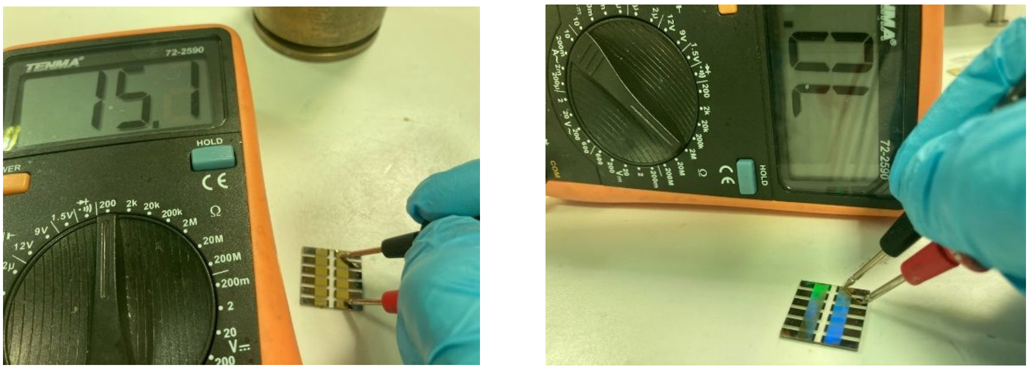

Figure S15 – Measuring the cathode sheet resistance (left) and the anode-cathode shunt resistance (right) for an etched BC-PSC with an L_E of 230 nm prior to nickel electrodeposition. A resistance of 15 Ω along the cathode rail corresponds to a sheet resistance of 4 Ω/sq, while the shunt resistance exceeds 2 MΩ.

Solubility of CuSCN based HTLs

To test the solubility of CuSCN HTLs, we measured out 20 mg of CuSCN (sigma Aldrich) each into 4 separate vials and then added 1 ml of various different solvents. The mixtures were shaken vigorously for 60 s to simulate the exposure to solvent during the perovskite spin coating step. The 4 solvents we tested were as follows: 0.5 ml of a 4:1 DMF:DMSO mixture with no additives, 0.5 ml of

DMF:DMSO with 461 mg/ml PbI_2, 0.5 ml DMF:DMSO with 172 mg/ml FAI, 0.5 ml DMSO with 390 mg/ml CsI. As can be seen on figure S16, CuSCN is not soluble in the first two mixtures, but dissolves rapidly in solutions containing FAI or CsI. This suggests that the FAI and CsI can assist the solubility of CuSCN, which means that the CuSCN is likely completely dissolved during the spin coating process. In addition to the rapid dissolution, a slow colour change from clear yellow to red is observed, which may be due to the degradation of the iodide salts into elemental iodine. This could explain why the CuSCN based BC-PSCs are even worse than devices with just gold.

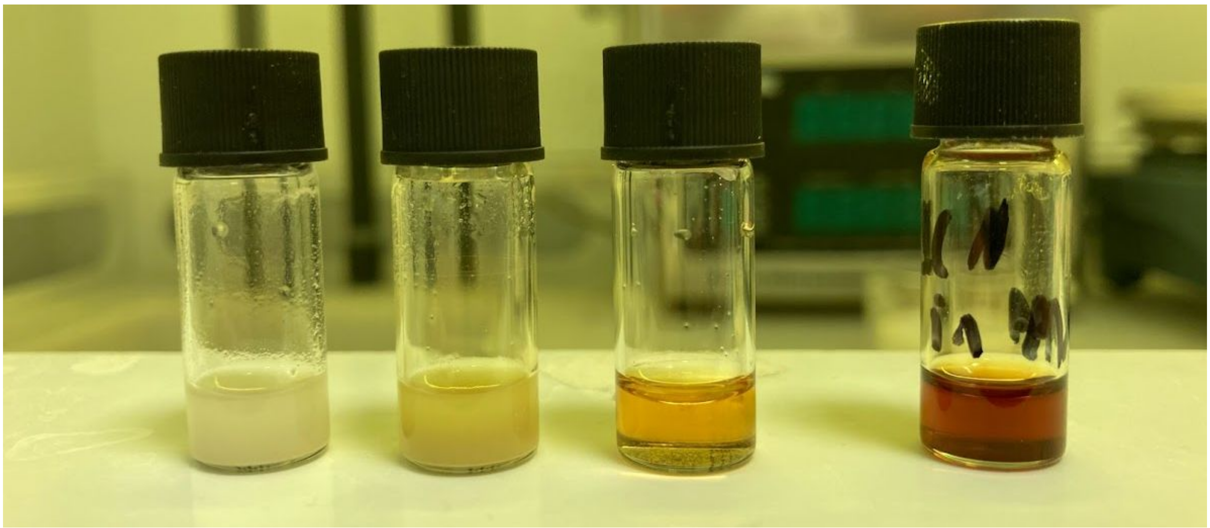

Figure S16 - Dissolving CuSCN in different solutions - left to right: 0.5 ml of a 4:1 DMF:DMSO mixture with no additives, 0.5 ml of DMF:DMSO with 461 mg/ml PbI_2, 0.5 ml DMF:DMSO with 172 mg/ml FAI, 0.5 ml DMSO with 390 mg/ml CsI.

Nanosphere Lithography

We spent considerable time trying to make honeycomb electrodes using nanosphere lithography, but like Bach and Coworkers we found that nanospheres smaller than a few microns do not lift off reliably, even after overnight sonication in chloroform. Below shows a Nickel grid made from 500 nm nanospheres plasma ashed for 8 minutes to reduce their size, and sonicated in chloroform overnight. Note that some of the spheres have lifted off, but most do not.

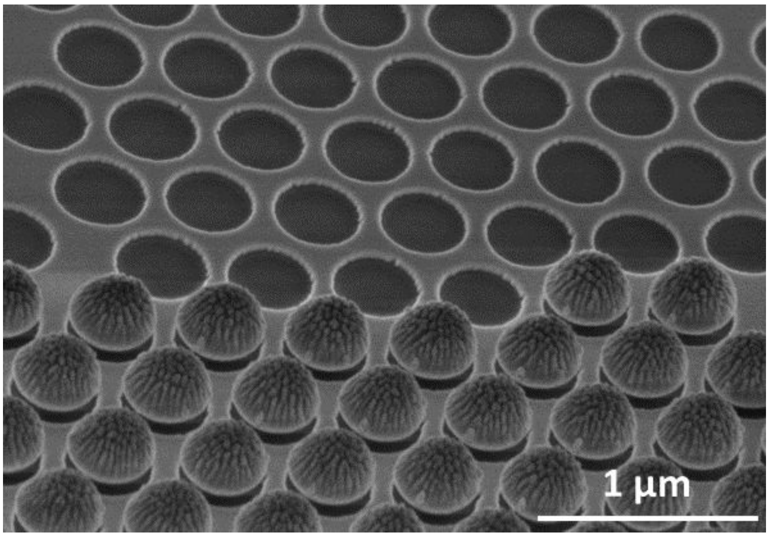

Figure S17 – Attempted nanosphere lithography using 500 nm spheres – only a partial lift off is achieved.

Dark Currents for Different Electrode Widths

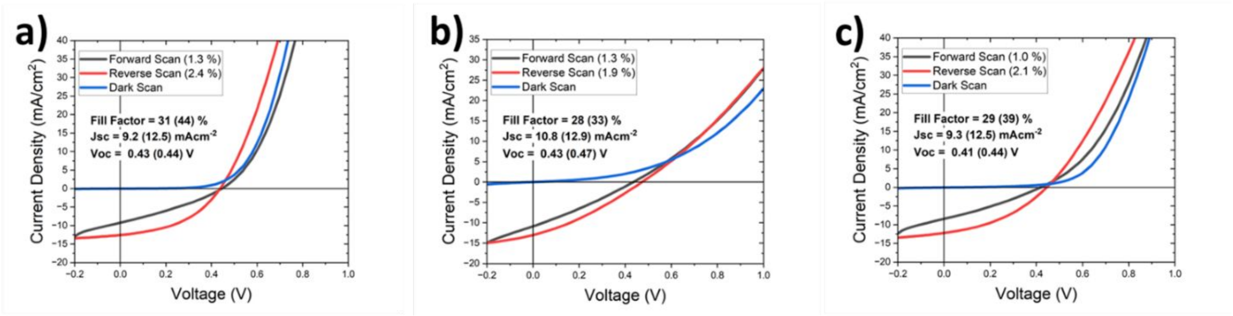

Figure S18 – Full J-V curves including dark currents for each of the devices shown in figure 5b). a) 230 nm width, b) 1000 nm width, c)2000 nm width

Future Upscaling Potential

Polymeric and Flexible Substrates

In mass production, it is desirable for the fabrication techniques to be compatible with flexible, polymeric substrates. The most common of these is polyethylene terephthalate (PET), which has a maximum operating temperature of 150 C. The Nanoimprint process itself can be operated at temperatures below 100 ◦C, and the high temperature insulating layer used here can be easily replaced with a low temperature material such as SU-8 epoxy, but the HTL is much more problematic. To be compatible with PET, a low temperature route to selectively depositing high quality hole transport layers is required, which has thus far not been achieved anywhere in the literature. However, pure polyimide substrates are commercially available under the brand "Kapton". These substrates are flexible, low cost, and can withstand temperatures as high as 400 ◦C. It is not normally used solar cells due to its poor transparency, however this is not an issue for a back contact device. Hence this production method could be easily adapted to flexible substrates in the future.

Impact of Sheet Resistance on Electrodeposition and Power Output

One of the main advantages of the back-contact structure is that it removes the need for transparent contacts, and therefore the sheet resistance of the electrodes can be substantially reduced. This is of particular importance for the electrodeposition, because ohmic voltage losses in the honeycomb grid would result in non-uniformity of the nickel deposit. Ohmic losses due to the sheet resistance are also known to significantly reduce the power conversion efficiencies of solar cells as they are scaled to large areas, and so it is important to consider the impacts these would have if our process were to be scaled up.

A completely accurate estimate of the voltage losses in the grid requires an iterative computer simulation that is beyond the scope of this article, but we can obtain a first-order estimate of the voltage loss using a simple "back of the envelope" calculation from ohm's law. We start by making the simple assumption of two parallel busbars separated by a distance **L**, each held at a constant voltage **V**, resulting in a current density leaving the plane of the device **J**. The pixel shall have a width denoted by **W**, but this ends up being irrelevant in the final calculation. We shall assume that J is uniform across the pixel for our calculation, but in reality it will change across the device as the

potential across the grid changes. Hence this is a "first order" calculation, where a real simulation would iterate by using the new voltage distribution to calculate the J distribution and then feed this back into a new voltage calculation. The first order approximation will give us an order of magnitude approximation for this effect, which give an idea of how big the problem is likely to be.

Current flows from the busbars towards the midpoint between them, decreasing linearly towards the midpoint as it charges leave out of the plane of the device. The total current flowing out of the device is shown in equation (1),

$$(1)\ \ Total\ Current\ Leaving\ the\ Plane = J \times W \times L$$

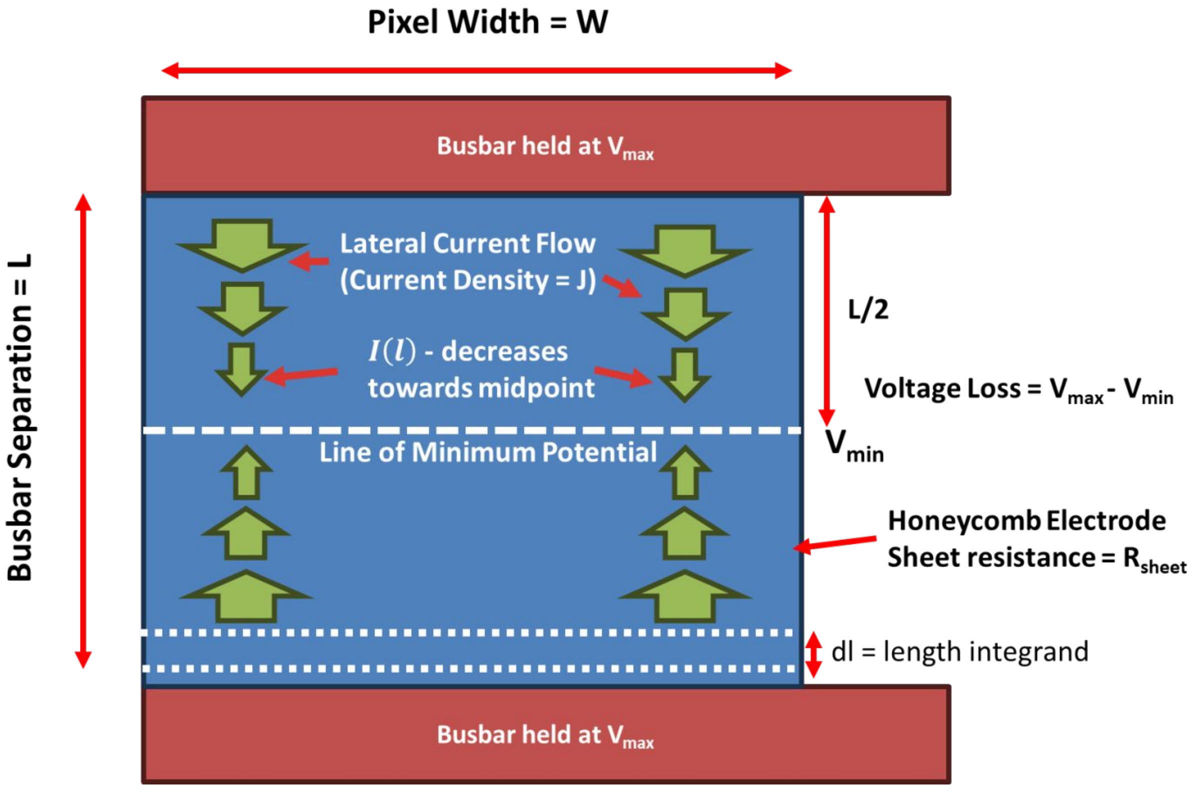

Figure S19 – Diagram of the simplified model used to calculate voltage loss. The key assumption is that $I(l)$ decreases linearly towards the midpoint due to a uniform distribution of current leaving the device.

We can divide the device into finite slices denoted by a width dl. Keeping the assumption of uniform current distribution, the current flowing through each of these slices a distance $\boldsymbol{l}$ from the bottom busbar is denoted by $\boldsymbol{I(l)}$, and can be represented by equation (2). The voltage loss across each of these elements, **dV**, can be simply calculated from ohm's law as shown on equation 3, where **dR** is the resistance of the slice. The resistance of the finite element can be calculated from the sheet resistance of the grid, denoted $\mathbf{R_{sheet}}$, from equation (4). Combining these expressions gives equation (5).

$$(2)\ I(l) = J \times W \times \left(\frac{L}{2} - l\right)$$

$$(3)\ dV = I(l) \times dR$$

$$(4)\ dR = R_{sheet} \times \frac{dl}{W}$$

$$(5)\ dV = J \times W \times \left(\frac{L}{2} - l\right) \times R_{sheet} \times \frac{dl}{W}$$

To get the maximum voltage loss, we now simply need to integrate this expression with the boundaries $l = 0$ and $l = \frac{L}{2}$, which results in equation (6), and can be solved to give the final equation (7).

$$(6)\ Voltage\ Loss = J \times R_{Sheet} \int_0^{L/2} \left(\frac{L}{2} - l\right) dl$$

$$(7)\ Voltage\ Loss = J \times R_{Sheet} \times \frac{L^2}{8}$$

In electrodeposition, current densities do not typically exceed 1 mA/cm^2, and a good target sheet resistance for the honeycomb electrode would be 1 Ω/sq. From this, if we were to assume a large busbar spacing of 10 cm, the total voltage loss, would be 12.5 mV, which is fairly low compared to the 0.55 V which we use for electrodeposition. Hence we can say with reasonable certainty that uniformity issues from electrodeposition are unlikely to be a major issue as long as the sheet resistance is good and the busbar spacing is low.

During device operation, the current density is expected to be much higher (at least 20 mA/cm^2), and plugging this value in gives a much larger voltage drop of 250 mV, which would correspond to a major loss in efficiency. Hence in a real device the busbars would need to be much closer together, and this shows that the effect of voltage losses during device operation will be a far greater concern than the voltage losses involved in electrodeposition. From these rough calculations, we conclude that the electrodeposition would not be a limiting step in the upscaling of back-contact solar cells.

Angled Evaporation as an Alternative to Electrodeposition

The most likely contributor to the poor efficiency of the BC-PSCs is the low quality NiOx layer. Better quality NiOx layers have in the past been deposited by room temperature electron beam evaporation[8], which would be ideal since it would result in higher quality NiOx layer and remove the high temperature annealing step. As mentioned previously, e-beam evaporation is not area-selective, but by performing the evaporation at a steep angle it may be possible to coat the gold electrode without filling in the holes (as shown below). This would require the depth of the holes ("h" in the diagram) to be at least the same as the electrode width ("L_E" in the diagram), but realistically an aspect ratio of 2:1 would probably be needed to get reliable results. This would be fairly feasible for the 230 nm period structures, but would become very problematic for L_E widths above 500 nm, as this would require a very high insulator thickness exceeding 1000 nm, which would result in a very long vertical diffusion length for electrons and would require an unfeasibly thick perovskite layer exceeding 1 micron. In addition, this technique would be extremely vulnerable to spot defects, as any missing pieces will create a bridge between anode and cathode that could short the device. Further work is needed to see if these issues can be overcome.

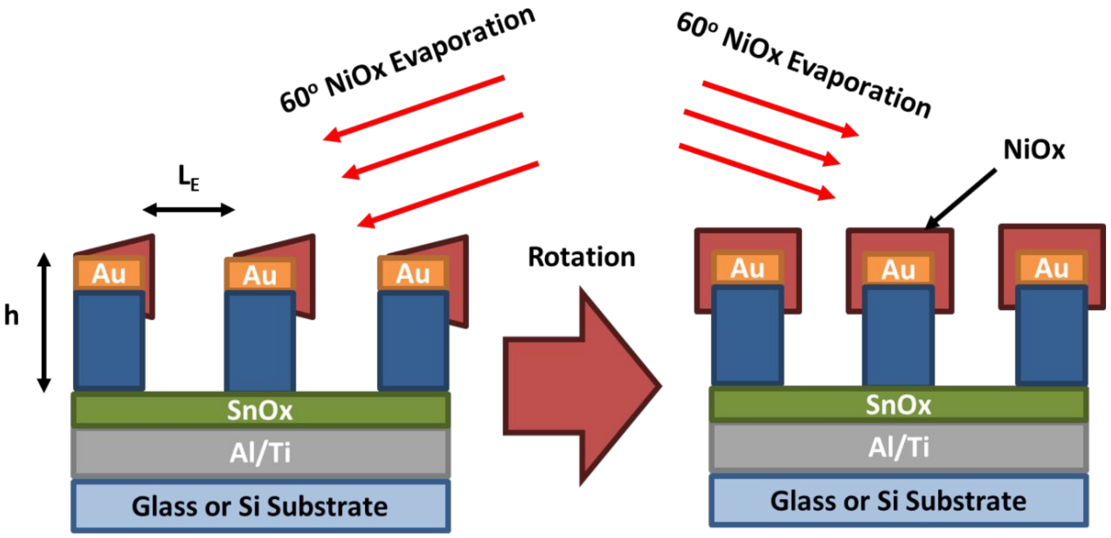

Figure S20 – A proposed method for angled evaporation to coat the electrodes. This would solve many of the problems described in this paper, but would require high aspect ratio structures to work well without causing parasitic deposition of the NiOx on the SnOx bottom layer.

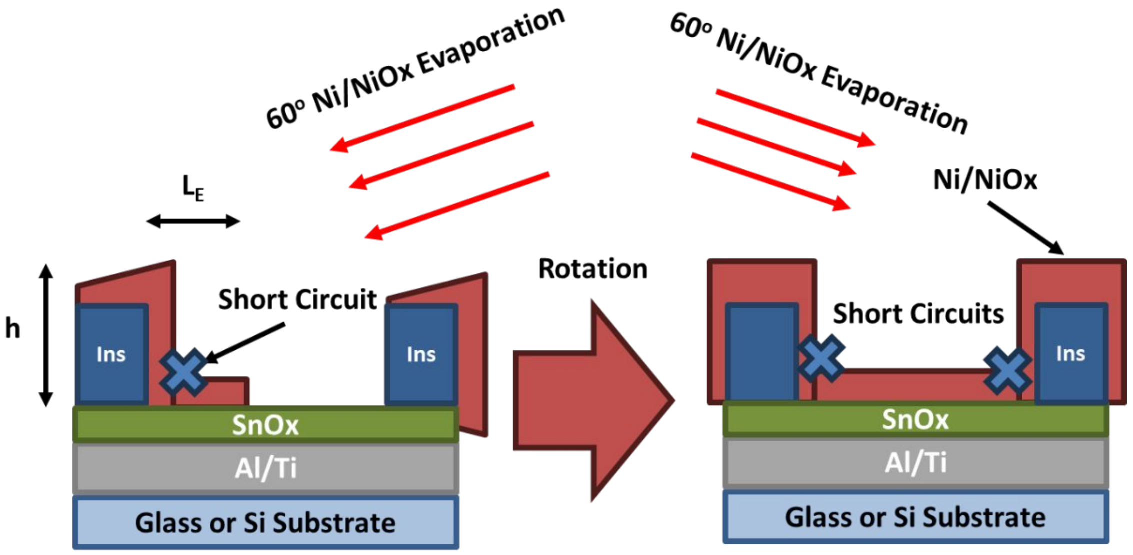

Figure S21 – The effect of spot defects on the angled evaporation technique – a missing pillar creates the possibility for a shorting pathway to occur.

Thickness Measurements of Perovskite

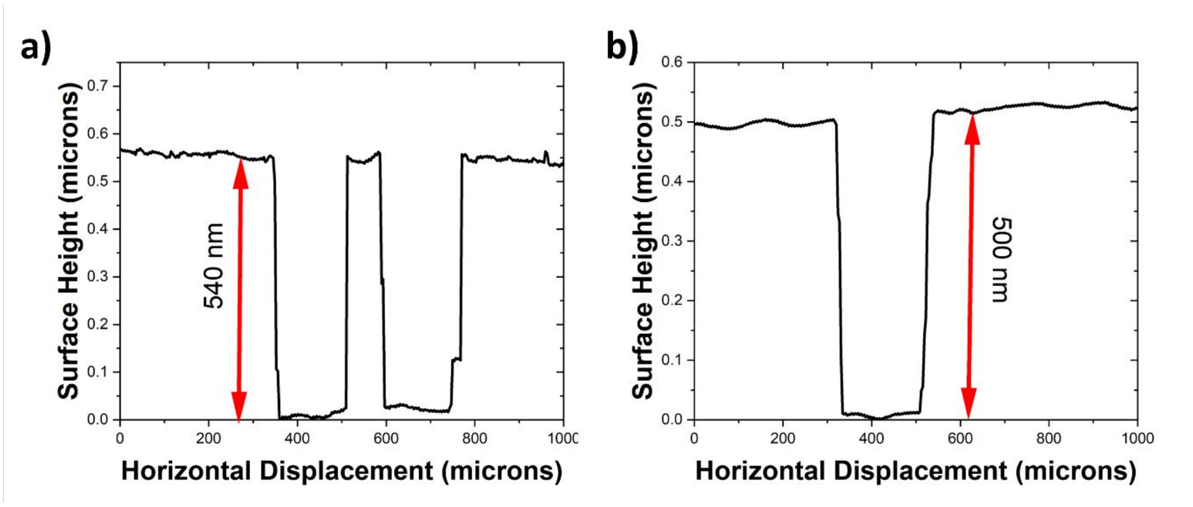

Figure 22 - Surface profilometry measurements of the CsMAFA film on a) A BC-PSC grid without the bottom electrode, and b) bare glass.

References

Yang Z, Yang W, Yang X, Greer J, Sheng J, Yan B, Ye J, "Optical Design and Optimisation for Back-Contact Perovskite Solar Cells", **Solar Energy**, 2020, **201**, 84-94, https://doi.org/10.1016/j.solener.2020.02.099

M. Goldiner and A. Vaysleb, "Mechanisms of Transition Metal Diffusion into Semiconductor Film" **MRS Online Proceedings Library,** 1993, **311**, 57–62, https://doi.org/10.1557/PROC-311-57

[2] Cheng N, Yu Z, Li W, Liu Z, Lei B, Zi W, Xiao Z, Tu Y, Gallegos D, "Highly efficient perovskite solar cells employing SnO_2 electron transporting layer derived from a tin oxalate precursor solution", **Journal of Power Sources**, 2022, **544**, 231871

[3] Han G, Lee S, Duff M, Qin F, Lee J, "Highly Bendable Flexible Perovskite Solar Cells on a Nanoscale Surface Oxide Layer of Titanium Metal Plates" ACS Appl. **Mater. Interfaces**, 2018, **10**, 4697−4704, https://doi.org/10.1021/acsami.7b16499

[4] Muthukrishnan A, Lee J, Kim J, Kim C, Jo S, "Low-temperature solution-processed SnO_2 electron transport layer modified by oxygen plasma for planar perovskite solar cells", **RSC Adv.**, 2022, **12**, 4883, https://doi.org/ 10.1039/d1ra08946c

[5] "Creating the perfect spiro layer" https://www.ossila.com/pages/perovskite-solar-cells#spiro-ometad (Accessed 08/05/2023)

[6] Ramachran K, Jeganathan C, Subbian K, "One-step electrodeposition of CuSCN/CuI nanocomposite and its hole transport-ability in inverted planar perovskite solar cells", **Nanotechnology**, 2021, **32**, 325402, https://doi.org/10.1088/1361-6528/abfe25

[7] Abziher T, Moghadamzadeh S, Shackmar F, Eggers H, Sutterluti F, Farooq A, Kojda D, Habnicht K, Schmager R, Mertens A, Paetzold U, "Electron-Beam-Evaporated Nickel Oxide Hole Transport Layers for Perovskite-Based Photovoltaics", Advanced Energy Materials, 2019, 9, 1802995, https://doi.org/10.1002/aenm.201802995
